# Supplementary material for: Rab4A-directed endosome traffic shapes pro-inflammatory mitochondrial metabolism in T cells via mitophagy, CD98 expression, and kynurenine-sensitive mTOR activation
Source: Nat Commun. 2024 Mar 22;15:2598. doi: 10.1038/s41467-024-46441-2 (PMC10960037; doi:10.1038/s41467-024-46441-2)
Supplement: Supplementary file 1 — Supplementary Information [file 41467_2024_46441_MOESM1_ESM.pdf]

SUPPLEMENTARY MATERIALS

List of Abbreviations: page 2.

Supplementary Tables S1-S4: pages 3-18.

Supplementary Figures S1-S19 with Legends: pages 19-58.

Supplementary References: page 59.

List of Abbreviations

ACLA, anticardiolipin antibodies;
ADP, adenosine diphosphate;
pAkt, pAkt^{Ser473}, Akt phosphorylated at serine residue 473;
ANA, anti-nuclear antibodies;
Anti-ApoH, anti-apolipoprotein H
Anti-β_2GPI, anti-β_2 glycoprotein I antibodies;
aPL, anti-phospholipid antibodies;
APS, anti-phospholipid syndrome;
B6, C57BL/6;
mClCCP, carbonyl cyanide m-chlorophenylhydrazone;
CMC, carboxymethyl cellulose;
$\Delta\psi_m$, mitochondrial transmembrane potential;
DAH, diffuse alveolar hemorrhage;
DHR, dihydrorhodamine;
DMEM, Dulbecco's modified Eagle medium;
EGTA, ethylene glycol-bis(2-aminoethylether)-N,N,N′,N′-tetraacetic acid;
ELISA, enzyme-linked immunosorbent assay;
ETC, electron transport chain;
FCCP, carbonyl cyanide-4-(trifluoromethoxy)phenylhydrazone;
HEPES, 4-(2-hydroxyethyl)-1-piperazineethanesulfonic acid;
HRP, horseradish peroxidase;
IFN, interferon;
Lpr, C57BL/6.lpr mice that carry a disrupted CD95 gene;
MHP, mitochondrial hyperpolarization;
MTG, MitoTracker green;
mTOR, mechanistic target of rapamycin;
mTORC1, mTOR complex 1;
mTORC2, mTOR complex 2;
OD, optical density;
OXPHOS, oxidative phosphorylation;
PBS, phosphate buffered saline;
P_i, inorganic phosphate;
PDBu, phorbol 12, 13-dibutyrate
PE, phosphatidylethanolamine;
PMSF, phenylmethanesulfonyl fluoride;
Pristane, 2, 6, 10, 14-tetramethylpentadecane;
PS, phosphatidylserine;
pS6K^{T389}, S6 ribosomal protein kinase phosphorylated at threonine residue 389;
pS6RP, pS6RP$^{S235/236}$, S6 ribosomal protein phosphorylated on serine residues 235 and 236
RB, respiration buffer;
RCR, respiratory control ratio;
ROI, reactive oxygen intermediate;
S6K, S6 ribosomal protein kinase;
SEM, standard error of the mean;
SDHA, succinate dehydrogenase complex flavoprotein subunit A of ETC complex II;
SLE, systemic lupus erythematosus;
TMB, 3,3′,5,5′-tetramethylbenzidine;
TMPD, N,N,N′,N′-tetramethyl-p-phenylenediamine

Supplementary Table S1. Mouse strain abbreviations.

Abbreviation	Mouse genotype	Rab4A modification	$CD4^{Cre}$
B6/WT	C57Bl/6	WT	
$B6/CD4^{Cre}$	$C57Bl/6.CD4^{Cre}$	WT	+
$B6/Rab4A^{Q72L}$	$C57Bl/6.Rab4A^{Q72L}$	$Rab4A^{Q72L\text{-}FL/FL}$	
$B6/Rab4A^{Q72L}$-KO	$C57Bl/6.Rab4A^{Q72L}$-$CD4^{Cre}$	$Rab4A^{Q72L\text{-}FL/FL}$ -$CD4^{Cre}$	+
B6.TC	C57Bl/6 SLE1.2.3. triple congenic	WT	
$B6.TC/CD4^{Cre}$	C57Bl/6 SLE1.2.3. triple congenic	WT	+
$B6.TC/Rab4A^{Q72L}$	C57Bl/6 SLE1.2.3. triple congenic	$Rab4A^{Q72L\text{-}FL/FL}$	
$B6.TC/Rab4A^{Q72L}$-KO	C57Bl/6 SLE1.2.3. triple congenic	$Rab4A^{Q72L\text{-}FL/FL}$ -$CD4^{Cre}$	+

Supplementary Table S2. ANTIBODIES USED FOR FLOW CYTOMETRY

Antibody reagents in this study were used at a 100-fold dilution.

Antibody target	Fluorochrome	Company	Cat. No.
CD3	BUV737	BD	564618
CD3	BV711	BD	563123
CD4	BUV496	BD	564667
CD4	PerCP-Cy5.5	BD	550954
CD8	APC-Cy7	Biolegend	100714
CD11b	PerCP-Cy5.5	BioLegend	101228
CD11b	BUV395	BD	563553
CD11c	BUV395	BD	744180
CD11c	AF488	Biolegend	117311
CD11c	PE-CF594	BD	562464
CD11c	BV605	BD	563057
CD19	APC-Cy7	BD	557655
CD19	AF700	Biolegend	115528
CD19	APC-R700	BD	565473
CD19	PerCP-Cy5.5	BD	551001
CD25	BUV395	BD	564022
CD38	PE-Cy7	Biolegend	102718
CD38	BV786	BD	740887
CD47	BV421	Biolegend	127527
CD51	BV650	BD	740546
CD61	BV510	BD	740117
CD68	PE-Cy7	Biolegend	137016
CD71	PE-Cy7	Biolegend	113812
CD98	PE	Invitrogen	12-0981-83
CD152	PE-Dazzle594	Biolegend	106318
D2DR	FITC	Alomone	ADR-002-F
DRD2	Alexa647	Santa Cruz	SC-5303
GLUT1	AF700	R&D	FAB1418N
GLUT4	AF647	R&D	FAB86541R
SERT	FITC	Alomone	AMT-004-F
CCR4	PE-Cy7	Biolegend	131214
CCR6	AF647	BD	557976
Gr1	PE-CF594	BD	562700
FoxP3	PerCP-Cy5.5	eBioscience	45-5773-82
Helios	AF647	Biolegend	137218
IFNy	AF647	Biolegend	505814
IL4	PE-Cy7	Biolegend	504118
IL9	PerCP-Cy5.5	Biolegend	514112
IL17a	PE/Dazzle594	Biolegend	506938
IL17a	AF488	Biolegend	506910
IL21	PE	eBioscience	12-7213-82
MTG	516nm	Invitrogen	M7514
pAKT	PE	BD	560378
pS6RP	AF488	Cell Signaling	4803S
TMRM	585nm	Invitrogen	M20036
FC block		BD	553142
FixPerm concentrate (10x)		eBioscience	00-5123-43
Fix Perm diluent		eBioscience	00-5223-56
Permeabilization buffer (10x)		eBioscience	00-8333-56

Supplementary Table S2, continued.

Antibody target	Fluorochrome	Company	Catalogue No
CD273/PD-L2	BUV563	BD	741431
TIGIT	BV421	BD	749781
PD-1	PE-Dazzle 594	Biolegend	135228
T-bet	eFluor 450	eBioscience	48-5825-82
Eomes	PerCP-eFluor710	eBioscience	46-4875-82

Isotype controls	Fluorochrome	Company	Catalogue No
Hamster IgG1	BV605	BD	563054
Hamster IgG1	BV510	BD	563197
Rat IgG1	BV650 º	BD	563848
Hamster IgG1	BV711	BD	563128
Rat IgG2a	BV786	BD	563335
Mouse IgG1	PE	BD	556650
Rat IgG2a	Brilliant Violet 421	Biolegend	400535
Mouse IgG2	Alexa Fluor 647	Biolegend	400234

Supplementary Table S3. ANTIBODIES USED FOR WESTERN BLOT

Primary antibody/Clone	Species/Isotype	Dilution	Manufacturer	Catalog number
4EBP1	Rabbit IgG	1/1000	Cell Signaling	9644
p4EBP1	Rabbit IgG	1/1000	Cell Signaling	2855
Actin	Mouse IgG1κ	1/10000	Millipore Sigma	MAB1501
AKT	Rabbit IgG	1/1000	Cell Signaling	4685
pAKT	Rabbit IgG	1/1000	Cell Signaling	4058S
Smooth muscle actin	mouse IgG2a	1/50	Dako	M0851
β-Catenin	mouse IgG1	1/1000	BD Biosciences	610154
Brdu	mouse IgG1	1/20	DSHB	G3G4
CD4	Rabbit IgG	1/1000	Santa Cruz	sc-7219
CD98	Rabbit IgG	1/1000	Thermofisher Scientific	PA5-96401
CRE	Rabbit IgG	1/10000	Millipore Sigma	69050-3
DRP1	Rabbit IgG	1/1000	Cell Signaling	8570
pDRP1	Rabbit IgG	1/1000	Cell Signaling	4494
Ecad	mouse	1/250	BD Biosciences	610182
FKBP12	mouse IgG1	1/1000	Fisher Scientific	BDB610808
HNF4	mouse IgG2a	1/300	R&D Systems	PP-H1415-OO
Ki67	mouse IgG1	1/250	BD Biosciences	556003
LAT1	Rabbit IgG	1/1000	Thermofisher Scientific	PA5-91842
mLST8	Rabbit IgG	1/1000	Cell Signaling	3274
mTOR	Rabbit IgG	1/1000	Cell Signaling	2983
Muc1	hamster	1/500	Neomarkers	HM-1630-P
P.H3	Rabbit IgG	1/100	Cell Signaling	9701
pMTOR	Rabbit IgG	1/1000	Cell Signaling	2971
Rab4A	Rabbit IgG	1/1000	Abcam	ab108974
Rab5A	Rabbit IgG	1/1000	Thermofisher Scientific	11947-1-AP
Rictor	Rabbit IgG	1/1000	Cell Signaling	2114
S6K	Mouse monoclonal IgG1 κ	1/1000	Santa Cruz	sc-8416
pS6K	Mouse monoclonal IgG1 κ	1/1000	Santa Cruz	sc-8418
Sox9	Rabbit IgG	1/500	Chemicon	AB5535
TSC1	Rabbit IgG	1/1000	Cell Signaling	4906
Tubulin	Rabbit IgG	1/1000	Cell Signaling	2128
YFP	goat	1/250	Abcam	ab6673

Secondary antibodies	Dilution	Manufacturer	Catalog number
Peroxidase-conjugated AffiniPure Goat Anti-Mouse IgG (H+L)	1/10000	Jackson ImmunoResearch	115-035-146
Anti-Rabbit IgG, peroxidase-linked species-specific F(ab')2 fragment (from donkey)	1/10000	Fisher Scientific	45-000-683

Supplementary Table S4. List of unique metabolites detected by a single 15-min targeted liquid chromatography–tandem mass spectrometry (LC–MS/MS) acquisition with a 3-ms dwell time and a 1.55-s duty cycle time. When available, KEGG and HMDB codes are listed for each compound.

Compounds	KEGG codes	HMDB codes
1,3-diphopshateglycerate	C00236	HMDB0001270
10-hydroxydecanoicacid	C02774	HMDB0000672
11-cis-eicosenoate	C04742	HMDB0003876
12,13-epoxy-9-hydroperoxy-10e-octadecenoate	C14832	HMDB0013623
13-carboxy-alpha-tocopherol	C02477	HMDB0012555
13-carboxy-gamma-tocopherol	C02483	HMDB0012557
13-hydroxy-alpha-Tocopherol	#N/A	HMDB0012559
13-hydroxy-alpha-tocotrienol	#N/A	HMDB0012560
15-hydroxy-(8z,11z,13e)-eicosatrienoate	C04742	HMDB0005045
1-deoxy-1-(n6-lysino)-d-fructose	#N/A	HMDB0062186
1-methyladenosine	C02494	HMDB0003331
1-methyl-histidine	C01152	HMDB0000001
1-methylnicotinamide	C02918	HMDB0000699
1-piperideine-6-carboxylate/1-piperideine-2-carboxylate	C00450	HMDB0062589
1-pyrroline-2-carboxylate/1-pyrroline-5- carboxylate	C03564	HMDB0006875
2,3-dihydroxybenzoic acid	C00196	HMDB0000397
2,3-diphosphoglyceric acid	C00236	HMDB0001294
2,3-epoxy-alpha-tocopheryl quinone/5,6-epoxy-alpha-tocopheryl quinone	C02477	HMDB0034408
2-acetamido-5-oxopentanoate	C01110	HMDB0006488
2-aminoacrylic acid	C02218	HMDB0003609
2-aminomuconate	C02220	HMDB0001241
2-aminooctanoic acid	#N/A	HMDB0000991
2-carboxy-2,3-dihydro-5,6- dihydroxyindole/l-dopaquinone	C00822	HMDB0039119
2-dehydro-d-gluconate	C06473	HMDB0011732
2-deoxy-d-ribose	C01801	HMDB0003224
2-deoxyglucose-6-phosphate	C06369	
2-hydroxy-2-methylbutanedioic acid	#N/A	HMDB0001844
2-hydroxyglutarate	C02630	HMDB0059655
2-isopropylmalic acid	C02504	HMDB0000402
2-keto-3-deoxy-d-glycero-d-galactononic acid 9-phosphate	#N/A	HMDB0001376
2-keto-3-deoxy-d-glycero-d-galactononic acid	#N/A	HMDB0001353
2-ketohaxanoic acid	#N/A	
2-keto-isovalerate	C00141	HMDB0000019
2-methylglutaconic acid	C03761	HMDB0002266
2-oxo-4-methylthiobutanoate	C01180	HMDB0001553
2-oxoadipate	C00322	HMDB0000225
2-oxobutanoate	C00109	HMDB0000005

2-oxoglutarate	C00940	HMDB0001552
2-phenylethanaminium	C05332	HMDB0012275
3(s)10(r)-oh-octadeca-6-trans-4,12-cis-trienoate	#N/A	
3(s)6(r)-dihydroxy-tetradec-8z- enoate/3(s)6(s)-dihydroxy-tetradec-8z-enoate	#N/A	
3,5-dihydroxy-3,4-dihydro-1,4- benzothiazine	#N/A	HMDB0062347
3alpha-7alpha-12alpha-25-Tetrahydroxy-12alpha-Trihydroxy-5beta-Cholestanoate 5beta-Cholestane-24-one/3alpha-7alpha-	#N/A	HMDB0062207
3-carboxy-alpha-chromanol	#N/A	HMDB0062349
3-d d-glucose	#N/A	
3-hydroxy-3-methylglutaryl-coa	C00356	HMDB0001375
3-hydroxyanthranilate	C00632	HMDB0001476
3-hydroxybutane-1,2,3-tricarboxylate/2-methylcitrate	C02225	HMDB0006471
3-hydroxybutyryl-coa	C01144	HMDB0001166
3-hydroxyhexadecanoylcarnitine	#N/A	HMDB0013336
3-methylcrotonoylglycine	#N/A	
3-methylphenylacetic acid	#N/A	HMDB0002222
3-oxo-10(r)-hydroxy-octadeca-6e8e12z- trienoate/3-oxo-10(s)-hydroxy-octadeca-6e8e12z-trienoate	#N/A	
3-oxo-6(r)-hydroxy-tetradec-8-cis- enoate/3-oxo-6(s)-hydroxy-tetradec-8z-enoate	#N/A	
3-oxo-8(r)-hydroxy-hexadeca-6e10z- dienoate/3-oxo-8(s)-hydroxy-hexadeca-6e10z-dienoate	#N/A	
3-phosphoglycerate	C00197	HMDB0000807
3-phospho-serine	C01005	HMDB0001721
3-s-methylthiopropionate	C08276	HMDB0031192
3-sulfino-l-alanine	C00606	HMDB0000996
3-sulfonatolactate	#N/A	HMDB0060176
4-(2-amino-3-hydroxyphenyl)-4-oxobutanoic acid o-glucoside	#N/A	HMDB0062374
4(r)-hydroxy-dodec-6z-enoate/4(s)-hydroxy-dodec-6z-enoate	#N/A	
4-(trimethylammonio)butanoate/acetylcholine	C01996	HMDB0000895
4alpha-5-Epoxy-8alpha-8alpha-Hydroperoxytocopherone Hydroperoxytocopherone/7-8-Epoxy-	#N/A	
4-aminobutyrate	C00334	HMDB0000112
4-hydroperoxy-2-nonenal	#N/A	HMDB0060287
4-hydroxy-2-nonenal/3,4-epoxynonanal	#N/A	
4-imidazolone-5-propanoate	C03680	HMDB0001014
4-nitrophenolate	C00870	HMDB0001232
4-nitrophenyl sulfate	#N/A	HMDB0006492
4-oxo-2-nonenal	#N/A	HMDB0060285
4-oxoproline	C01877	
4-pyridoxic acid	C00847	HMDB0000017
4-trimethylammoniobutanal	C01149	HMDB0001345
5,6-dihydrothymine	C00906	HMDB0000079

5-amino-1-(5-phospho-d-ribosyl)imidazole	C04751	HMDB0062575
5-guanidino-2-oxopentanoic acid	#N/A	HMDB0004225
5-hydroxyindoleacetate	C05635	HMDB0000763
5-hydroxyisourate	C11821	HMDB0030097
5-methoxyindoleacetate	C05660	HMDB0004096
5-methoxytryptophan	C00643	HMDB0002339
5-methyl-thf	C00440	HMDB0001354
5-phospho-beta-d-ribosylamine	C03090	HMDB0001128
5-phosphoribosyl-1-pyrophosphate	C00119	HMDB0000280
5-thio-glucose, is	#N/A	
6(s)-hydroxy-tetradeca-2e4e8z-trienoate trienoate/6(r)-hydroxy-tetradeca-2e4e8z-	#N/A	
6-phospho-d-gluconate	C00345	HMDB0001316
6-phospho-d-glucono-1,5-lactone	C01236	HMDB0001127
7,8-dihydrofolate	C00415	HMDB0001056
7-methylguanosine	C20674	HMDB0001107
8(r)-hydroxy-hexadeca-2e4e6e10z-2e4e6e10z-tetraenoate tetraenoate/8(s)-hydroxy-hexadeca-	#N/A	
9(10)-epome/12(13)-epome	#N/A	HMDB0004701
9,10-hydroxyoctadec-12(z)-enoate/12,13-hydroxyoctadec-9(z)-enoate	#N/A	HMDB0004705
Acadesine	C04663	
Acetoacetate	C00164	HMDB0000060
Acetoacetyl-coa	C00332	HMDB0001484
Acetyl adenylate	C05993	HMDB0006880
Acetylcarnitine DL	C02571	HMDB0000201
Acetyl-coa	C00024	HMDB0001206
Acetyllysine	C12989	HMDB0000446
Acetylphosphate	C00227	HMDB0001494
Aconitate	C00417	HMDB0000072
Adenine	C00147	
Adenosine	C00212	HMDB0000050
Adenosine 5-phosphosulfate	C00224	HMDB0001003
Adipic acid	C06104	HMDB0000448
ADP	C00008	HMDB0001341
ADP-d-glucose	C00498	HMDB0006557
Adrenaline/l-normetanephrine	C00788	HMDB0000819
Agmatinium	C00179	HMDB0001432
A-ketoglutarate	C00026	HMDB0059655
AMP	C00020	HMDB0003540
Anthranilate	C00108	HMDB0001123
Arachidonate/eicosatetranoic acid	C00219	HMDB0001043
Argpyrimidine	#N/A	HMDB0037180
Ascorbic acid	C00072	HMDB0000044
Asparagine	C00152	HMDB0000168
Aspartate	C00049	HMDB0000191

ATP	C00002	HMDB0000538
Atrolactic acid	#N/A	
Azelaic acid	C08261	HMDB0000784
Benzoate	C00180	HMDB0001870
Beta-carboline	C20157	HMDB0012897
Beta-hydroxy-beta-methylbutyrate	C20827	HMDB0000754
Betaine	C00719	HMDB0000043
Betaine aldehyde	C00576	HMDB0001252
Biotin	C00120	HMDB0000030
But-2-ene-1,2,3-tricarboxylate	C04225	HMDB0006357
Butyryl-coa	C00136	HMDB0001088
C10h16o(3)	C09847	HMDB0035092
C14h24o3(4)	#N/A	HMDB0032369
C16h26o3(4)	C09694	HMDB0060055
C18h32o4(3)	C07338	HMDB0062637
C20h28o2(7)	C00777	HMDB0038702
C20h30o2(4)	C06428	HMDB0036826
C20h30o3(6)	C00909	HMDB0035311
C20h30o4(18)	C05957	HMDB0041055
C20h30o5(11)	C20214	HMDB0038693
C20h32o3(12)	C14749	HMDB0038693
C20h32o4(21)	C02165	HMDB0062756
C20h32o5(15)	C06314	HMDB0062798
C20h32o6(8)	C05956	HMDB0062683
C20h34o4(4)	C14774	HMDB0035379
C27h42o4(3)	C08902	HMDB0030066
C27h44o2(5)	C01561	HMDB0035585
C27h44o3(5)	C17336	HMDB0060133
C27h46o3(6)	C06341	HMDB0062675
C27h46o4(3)	C01301	HMDB0062208
C3h4o2(3)	C00511	HMDB0031647
C3h6o2(4)	C00163	HMDB0031229
C4h8o3(3)	C00989	HMDB0031212
C5h10o5(6)	C00121	HMDB0000751
C6h11no3(4)	C02946	HMDB0094698
C8h16no9p(4)	C00357	HMDB0006480
Caprate	#N/A	HMDB0000511
Carbamoyl phosphate	C00169	HMDB0001096
Carnitine	C00487	HMDB0000062
Carnosine	C00386	HMDB0000033
CDP	C00112	HMDB0001546
CDP-choline	C00307	HMDB0001413
CDP-ethanolamine	C00570	HMDB0001564
Cellobiose	C00185	HMDB0000055
Cervonic acid c22:6 n-3/docosahexaenoate	C06429	HMDB0062579
Cholesteryl sulfate	C18043	HMDB0000653

Cholic acid	C00695	HMDB0000619
Choline	C00114	HMDB0000097
Choline phosphate	C00588	HMDB0001565
Citraconic acid	C02226	HMDB0000634
Citrate	C00158	HMDB0000094
Citrate-isocitrate	C00311	HMDB0000094
Citrulline	C00327	HMDB0000904
Clupanodonic acid/docosa-4,7,10,13,16- pentaenoic acid	#N/A	HMDB0006528
CMP	C00055	HMDB0000095
CMP-2-aminoethylphosphonate	C05673	HMDB0060067
CMP-n-acetyl-beta-neuraminate	C00128	HMDB0062695
CMP-n-trimethyl-2- aminoethylphosphonate	C05674	HMDB0060072
Coenzyme A	C00010	HMDB0001423
Creatine	C00300	HMDB0000064
Creatinine	C00791	HMDB0000562
CTP	C00063	HMDB0000082
Cyclic ADP-ribose	C13050	HMDB0011671
Cyclic bis(3->5) dimeric GMP	C16463	HMDB0001314
Cyclic-AMP	C00575	HMDB0000058
Cystathionine	C02291	HMDB0000099
Cysteine	C00097	HMDB0000574
Cysteine sulfonic acid	C00606	HMDB0000731
Cystine	C00491	HMDB0000192
Cytidine	C00475	HMDB0000089
Cytosine	C00380	HMDB0000630
D-alanyl-d-alanine	C00993	HMDB0003459
dAMP	C00360	HMDB0000905
dATP	C00131	HMDB0001532
dCDP	C00705	HMDB0001245
dCMP	C00239	HMDB0001202
dCTP	C00458	HMDB0000998
Deoxyadenosine	C00559	HMDB0000101
Deoxycholic acid	C04483	HMDB0000626
Deoxyguanosine	C00330	HMDB0000085
Deoxyinosine	C05512	HMDB0000071
Deoxyribose-phosphate	C00673	HMDB0001031
Deoxyuridine	C00526	HMDB0000012
Dephospho-coa	C00882	HMDB0001373
D-erythronic acid	#N/A	HMDB0000613
D-erythrose-4-phosphate	C00279	HMDB0001321
dGDP	C00361	HMDB0000960
D-glucarate	C00818	HMDB0000663
D-gluconate	C00257	HMDB0000625
D-glucosamine-1-phosphate	C06156	HMDB0001109
D-glucosamine-6-phosphate	C00352	HMDB0001254

D-glucuronate 1-phosphate	C05385	HMDB0003976
D-glyceraldehdye-3-phosphate	C00118	HMDB0001112
dGMP	C00362	HMDB0001044
dGTP	C00286	HMDB0001440
Dihomo-gamma-linolenic acid (n-6)	C03242	HMDB0002925
Dihydroorotate	C00337	HMDB0003349
Dihydroxy-acetone-phosphate	C00111	HMDB0001473
Diiodothyronine	#N/A	HMDB0000582
Dimethylglycine	C01026	HMDB0000092
Diphosphate	C00013	HMDB0000250
Dl-pipecolic acid	C00408	HMDB0000070
Dodecanedioic acid	C02678	HMDB0000623
Dopaminium	C03758	HMDB0000073
Dopaminochrome	C03758	HMDB0000073
D-sedoheptulose-7-phosphate	C00447	HMDB0001068
dTDP	C00363	HMDB0001274
dTMP	C00364	HMDB0001227
dTTP	C00459	HMDB0001342
dUMP	C00365	HMDB0001409
dUTP	C00460	HMDB0001191
Elaidic carnitine/vaccenyl carnitine	#N/A	HMDB0006464
Erythro-5-hydroxy-l-lysinium	C16741	HMDB0062570
Estrone 3-sulfate	C02538	HMDB0001425
Ethamp[C]	#N/A	
Ethanolamine	C00189	HMDB0000149
FAD	C00016	HMDB0001248
Flavone	#N/A	HMDB0003075
FMN	C00061	HMDB0001520
Folate	C00504	HMDB0000121
Fructose-1,6-bisphosphate	C05378	HMDB0001058
Fructose-6-phosphate	C00085	HMDB0000124
Fructoseglycine	#N/A	HMDB0060278
Fumarate	C00122	HMDB0000134
Galactosylglycerol	C05401	HMDB0006790
Gamma-l-glutamyl-l-cysteine	C00669	HMDB0001049
GDP	C00035	HMDB0001201
GDP-alpha-d-mannose	C00096	HMDB0001163
Geranyl-PP	C00341	HMDB0001285
Glucono-d-lactone	C00198	HMDB0000150
Glucosamine	C00329	HMDB0001514
Glucose, neg	C00031	HMDB0000122
Glucose-1-phosphate	C00103	HMDB0001586
Glucose-6-phosphate	C00668	HMDB0001401
Glutamate	C00025	HMDB0000148
Glutamine	C00064	HMDB0000641
Glutathione	C00051	HMDB0000125

Glutathione disulfide	C00127	HMDB0003337
Glycerate	C00258	HMDB0000139
Glycerophosphocholine	C00670	HMDB0000086
Glycine	C00037	HMDB0000123
Glycocholate	C01921	HMDB0000138
Glycolate	C00160	HMDB0000115
Glyoxylate	C00048	HMDB0000119
GMP	C00144	HMDB0001397
GTP	C00044	HMDB0001273
Guanidoacetic acid	C00581	HMDB0000128
Guanine	C00242	HMDB0000132
Guanosine	C00387	HMDB0000133
Guanosine 5-diphosphate,3-diphosphate	C01228	HMDB0059638
Heptadecanoyl carnitine	#N/A	HMDB0006210
Hexadecanedioc acid	#N/A	HMDB0000220
Hexose-phosphate	0	
Histamium	C00388	HMDB0000870
Histidine	C00135	HMDB0000177
Histidinol	C00860	HMDB0003431
Homocysteic acid	C16511	HMDB0002205
Homocysteine	C05330	HMDB0000742
Homoserine	C00263	HMDB0000719
Homovanillate/3-(4- hydroxyphenyl)lactate/3-methoxy-4-hydroxyphenylglycolaldehyde	#N/A	HMDB0000118
Hydroxyisocaproic acid	0	HMDB0000746
Hydroxyphenylacetic acid	C05852	HMDB0000440
Hydroxyphenylpyruvate	C01179	HMDB0011663
Hydroxyproline	C01157	HMDB0000725
Hypotaurine	C00519	HMDB0000965
Hypoxanthine	C00262	HMDB0000157
IDP	C00104	HMDB0003335
Imidazole	C01589	HMDB0001525
Imidazole-4-acetaldehyde	C05130	HMDB0003905
Imidazoleacetic acid	C02835	HMDB0002024
IMP	C00130	HMDB0011681
Indole	C00463	HMDB0000738
Indole-3-acetate/(5-hydroxyindol-3-yl)acetaldehyde	C00954	HMDB0000197
Indole-3-carboxylic acid	C19837	HMDB0003320
Indoleacrylic acid	C00331	HMDB0000734
Inosine	C00294	HMDB0000195
Inositol trisphosphate	C01245	HMDB0000189
Isocitrate	C00311	HMDB0000193
Isoputreanine/putreanine	#N/A	HMDB0006009
Kynurenic acid	C01717	HMDB0000715
Kynurenine	C00328	HMDB0000684
L-2-amino-3-oxobutanoic acid	C03508	HMDB0006454

L-3-cyanoalanine/5,6-dihydrouracil	C02512	HMDB0060245
Lactate	C00186	HMDB0000190
Lactose	C00243	HMDB0000186
L-arabinitol/xylitol/d-ribitol	C00532	HMDB0001851
L-arginino-succinate	C03406	HMDB0000052
Laurate	#N/A	
L-cysteate	C00506	HMDB0002757
L-cysteine	C00097	HMDB0000574
L-cysteinylglycine	C01419	HMDB0000078
L-erythrulose	C02045	HMDB0006293
Leucine-isoleucine	C00123	HMDB0000172
L-fucose/l-fuculose	C01019	HMDB0000174
L-glutamine	C00064	HMDB0000641
L-iditol/d-glucitol/galactitol	C01507	HMDB0011632
Limonene/(+)-alpha-pinene	C06099	HMDB0004321
Linoelaidic acid (all trans c18:2)/linoleate/octadecadienoate (n-c18:2)	C01595	HMDB0000673
Linoleyl carnitine/linoelaidyl carnitine	#N/A	HMDB0006469
Lipoamide	C00248	HMDB0000962
Lipoate	C00725	HMDB0000962
L-palmitoylcarnitine	C02990	HMDB0000222
L-prolinylglycine/glycylproline	#N/A	HMDB0011178
L-threonate	C01620	HMDB0062620
L-xylonate/l-lyxonate	C05411	HMDB0060255
Lysine	C00047	HMDB0000182
Malate	C00149	HMDB0031518
Maleic acid	C01384	HMDB0000176
Malonate/3-hydroxypyruvate	C00383	HMDB0000691
Malondialdehyde	C19440	HMDB0006112
Malonyl-coa	C00083	HMDB0001175
Margarate	#N/A	HMDB0002259
Methionine	C00073	HMDB0000696
Methionine sulfoxide	C02989	HMDB0002005
Methyl indole-3-acetate	C20635	HMDB0029738
Methylcysteine	C00155	HMDB0002108
Methylmalonic acid	C02170	HMDB0000202
Methylnicotinamide	C02918	HMDB0003152
Mevalonate	C00418	HMDB0000227
Myo-inositol	C00137	HMDB0000211
Myristate	C06424	HMDB0000806
N(6)-carboxyethyllysine	#N/A	
N(6)-carboxymethyllysine	#N/A	
N(8)-acetylspermidinium/n(1)-acetylspermidinium	#N/A	HMDB0002189
N2-formyl-n1-(5-phospho-d-ribosyl)glycinamide	#N/A	HMDB0001308
N6-acetyl-l-lysine	C02727	HMDB0000206
N6n6n6-trimethyl-l-lysine	#N/A	HMDB0001325
NAADP	C13051	HMDB0001179

N-acetylaspartic acid	#N/A	HMDB0000812
N-acetylgalactosamine sulfate	#N/A	HMDB0000841
N-acetyl-glucosamine	C00140	HMDB0000215
N-acetyl-glucosamine-1-phosphate	C04256	HMDB0001367
N-acetyl-glutamate	C00624	HMDB0001138
N-acetyl-glutamine	N/A	HMDB0006029
N-acetyl-l-alanine	C00624	HMDB0000766
N-acetyl-l-aspartate/2-amino-3-oxoadipate	C01042	HMDB0000812
N-acetyl-l-cysteine	C06809	HMDB0001890
N-acetyl-l-ornithine	C00437	HMDB0003357
N-acetylmethionine	C02712	HMDB0011745
N-acetylneuraminate	C00270	HMDB0000230
N-acetylputrescine	C02714	HMDB0002064
N-acetyl-seryl-aspartate	#N/A	HMDB0062501
NAD+	C00003	HMDB0000902
NADH	C00004	HMDB0001487
NADP+	C00006	HMDB0000217
NADPH	C00005	HMDB0000221
Naphthalene	C00829	HMDB0029751
N-benzoylglycinate/adrenochrome	#N/A	HMDB0000714
N-carbamoyl-l-aspartate	C00438	HMDB0000828
N-formimidoyl-l-glutamate/n-acetyl-asparagine	#N/A	HMDB0000854
N-formylanthranilate/noradrenochrome	#N/A	HMDB0004089
Ng,Ng-dimethyl-l-arginine	C03626	HMDB0001539
Nicotinamide	C00153	HMDB0001406
Nicotinamide ribotide	C00455	HMDB0000229
Nicotinate	C00253	HMDB0001488
N-methyl-4,6,7-trihydroxy-1,2,3,4-tetrahydroisoquinoline	#N/A	HMDB0060065
N-methylethanolaminium phosphate	C01210	HMDB0060173
Nonanoate	C01601	HMDB0000847
N-phosphocreatinate	C02305	HMDB0062567
O-acetyl-l-serine	C00979	HMDB0003011
Octulose-1,8-bisphosphate (obp)	#N/A	
Octulose-monophosphate (o8p-o1p)	#N/A	
Omega hydroxy dodecanoate (n-c12:0)	#N/A	HMDB0002059
Omega hydroxy hexadecanoate (n-c16:0)	#N/A	HMDB0006294
Omega hydroxy tetradecanoate (n-c14:0)	#N/A	
O-methylhippurate	#N/A	HMDB0000859
Ophthalmic acid	#N/A	HMDB0005765
Ornithine	C00077	HMDB0000214
Orotate	C00295	HMDB0000226
Orotidine-5-phosphate	C01103	HMDB0000218
Oxalate	C00209	HMDB0002329
Oxalatosuccinate	C05379	HMDB0003974
Oxaloacetate	C00036	HMDB0000223
Palmitate	C00249	HMDB0000220

Palmitoleate	C08362	HMDB0003229
P-aminobenzoate	C00568	HMDB0001392
Pantothenate	C00864	HMDB0000210
Pendtadenoyl carnitine	#N/A	
Pentadecanoate	C16537	HMDB0000826
Pentosidine	C21014	HMDB0003933
Perillic acid	C11924	HMDB0004586
Perillyl aldehyde	C02576	HMDB0003647
Phenylacetaldehyde	C00601	HMDB0006236
Phenylacetate/(4-hydroxyphenyl)acetaldehyde	#N/A	HMDB0003767
Phenylalanine	C00079	HMDB0000159
Phenyllactic acid	C01479	HMDB0000779
Phenylpropiolic acid	C05629	HMDB0002359
Phenylpyruvate	C00166	HMDB0000205
Phosphodimethylethanolamine	C13482	HMDB0060244
Phosphoenolpyruvate	C00074	HMDB0000263
Phosphorylcholine	C00588	HMDB0001565
P-hydroxybenzoate	C00156	HMDB0000500
Phytanate/arachidate	#N/A	HMDB0000801
Prephenate	C00254	HMDB0012283
Pristanic acid/pristanate	#N/A	HMDB0000795
Proline	C00148	HMDB0000162
Propionyl-coa	C00100	HMDB0001275
Pseudoecgonine/ecgonine	#N/A	HMDB0006548
Purine	C15587	HMDB0001366
Putrescine	C00134	HMDB0001414
Pyridoxal	C00018	HMDB0001545
Pyridoxamine	C00534	HMDB0001431
Pyridoxine	C00314	HMDB0000239
Pyroglutamic acid	C01879	HMDB0000267
Pyrophosphate	C00013	HMDB0000250
Pyrrolines	#N/A	HMDB0012497
Pyruvate	C00022	HMDB0000243
Quinolinate	C03722	HMDB0000232
Riboflavin	C00255	HMDB0000244
Ribose-phosphate	C00117	HMDB0001548
S-[2-carboxy-1-(1 h-imidazol-4-yl)ethyl]-l-cysteine	#N/A	
S-adenosyl-l-homocysteine	#N/A	HMDB0000939
S-adenosyl-l-methioninamine	C01137	HMDB0000988
S-adenosyl-l-methionine	C00019	HMDB0001185
Salsolinol 1-carboxylate	#N/A	HMDB0013068
Sarcosine	C00213	HMDB0000271
Sebacic acid	#N/A	HMDB0000792
Sedoheptitol	#N/A	
Sedoheptulose 1,7-bisphosphate (sbp)	#N/A	HMDB0060274
Serine	C00065	HMDB0000187
Serotonin	C00780	HMDB0000259
Shikimate	C00493	HMDB0003070
Shikimate-3-phosphate	C03175	

S-lactoylglutathionate	#N/A	HMDB0001066
S-methyl-5-thioadenosine	C00170	HMDB0001173
Sn-glycerol-3-phosphate	C00093	HMDB0000126
Sorbitol	#N/A	HMDB0000247
Spermidine	C00315	HMDB0001257
Spermidine dialdehyde-1	#N/A	HMDB0013076
Spermidine dialdehyde-2	#N/A	HMDB0013076
Spermine	C00750	HMDB0001256
Sphingosine/(2s)-1-hydroxy-3-oxooctadecan-2-aminium	#N/A	HMDB0000252
Sphingosine-5-P	#N/A	HMDB0000277
S-ribosyl-l-homocysteine	#N/A	HMDB0002238
Stearate	C01530	HMDB0000827
Stearoylcarnitine	#N/A	HMDB0000848
Suberic acid	C08278	HMDB0000893
Succinate	C00042	HMDB0000254
Succinyl carnitine	#N/A	HMDB0061717
Succinyl-coa	C00091	HMDB0001022
Succinyl-coa-methylmalonyl-coa	#N/A	
Sulfate derivative of norepinephrine	#N/A	
Sulfoglycolithocholate	#N/A	HMDB0062801
Taurine	C00245	HMDB0000251
Taurocholic acid	#N/A	HMDB0000036
Taurodeoxycholic acid	C05463	HMDB0000896
Taurolithocholate	#N/A	HMDB0000896
Tetradecenoate (N-C14:1)	#N/A	HMDB0000499
Thiamine	C00378	HMDB0000235
Thiamine pyrophosphate	C00068	HMDB0001372
Thiamine-phosphate	C01081	HMDB0002666
Thiosulfate	#N/A	HMDB0000257
Threonine	C00188	HMDB0000167
Thymidine	C00214	HMDB0000273
Thymine	C00178	HMDB0000262
Trans, trans-farnesyl diphosphate	C00448	HMDB0000961
Trans-4,5-epoxy-2(e)-decenal	#N/A	HMDB0013105
Trans-hexadec-2-enoyl carnitine	#N/A	HMDB0006317
Trans-vaccenate/elaidate/oleate	#N/A	HMDB0003231
Trehalose-6-phosphate	C00689	HMDB0001124
Trehalose-sucrose	C00089	
Tryptophan	C00525	HMDB0030396
Tyramine o-sulfate	#N/A	HMDB0006409
Tyraminium	C00483	HMDB0062629
Tyrosine	C00082	HMDB0000158
UDP	C00015	HMDB0000295
UDP-alpha-d-xylose	C00190	HMDB0001018
UDP-d-glucose	C00029	HMDB0000286
UDP-d-glucuronate	C00167	HMDB0000935
UDP-N-acetyl-glucosamine	C00043	HMDB0000290
UMP	C00105	HMDB0060282
Uracil	C00106	HMDB0000300

Urea	C00086	HMDB0000294
Uric acid	C00366	HMDB0000289
Uridine	C00299	HMDB0000296
Urocanate	C00785	HMDB0000301
UTP	C00075	HMDB0000285
Valine	C00183	HMDB0000883
Xanthine	C00385	HMDB0000292
Xanthosine	C01762	HMDB0000299
Xanthosine-5-phosphate	C00655	HMDB0001554
Xanthurenic acid	C02470	HMDB0000881

SUPPLEMENTARY FIGURES

Figure S1

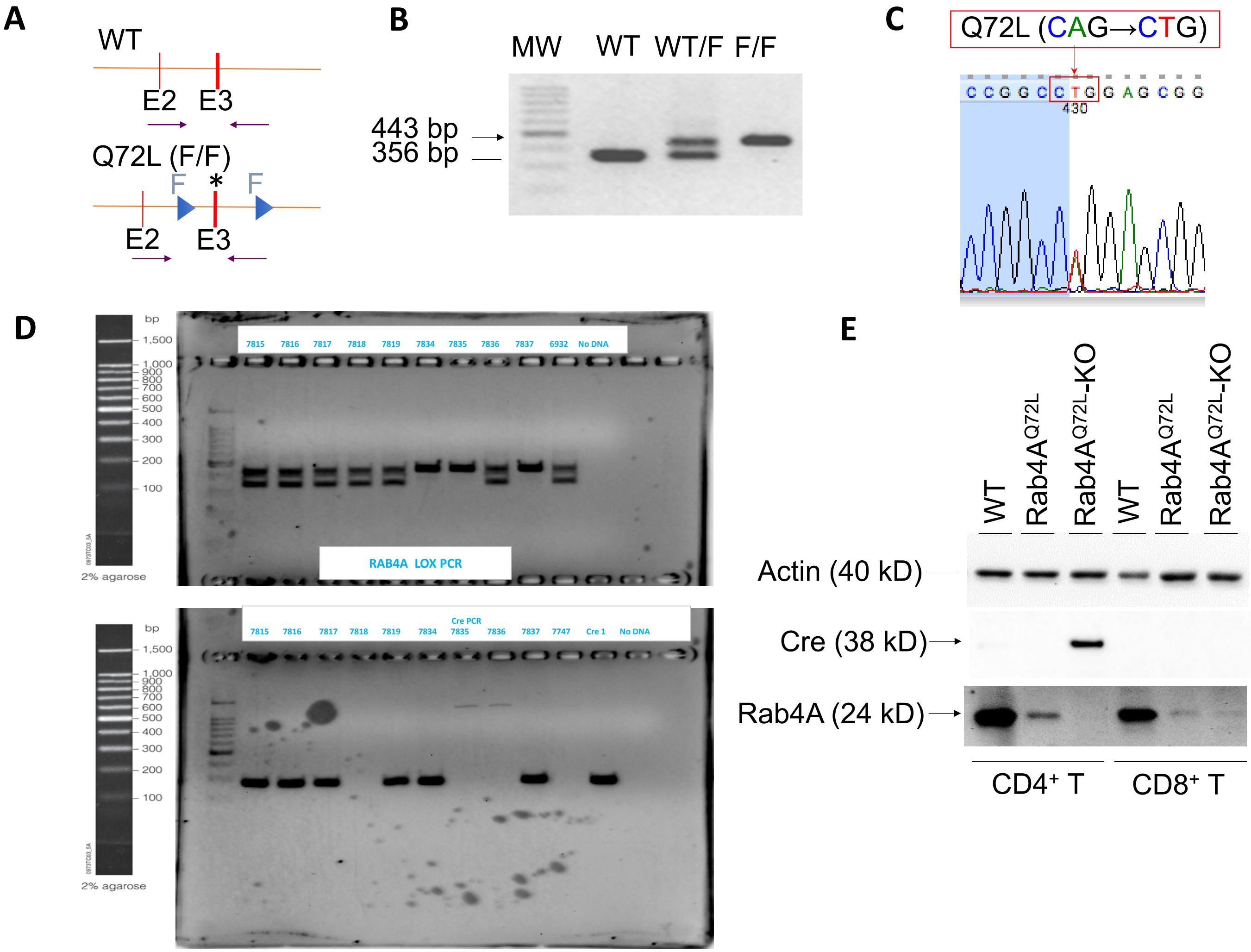
A
WT
E2
E3
Q72L (F/F)
F
*
F
E2
E3
B
MW
WT
WT/F
F/F
443 bp
356 bp
C
Q72L (CAG→CTG)
C C G G C C T G G A G C G G
430
D
bp
1,500
1,000
900
800
700
600
500
400
300
200
100
2% agarose
7815 7816 7817 7818 7819 7834 7835 7836 7837 6932 No DNA
RAB4A LOX PCR
bp
1,500
1,000
900
800
700
600
500
400
300
200
100
2% agarose
Cre PCR
7815 7816 7817 7818 7819 7834 7835 7836 7837 7747 Cre 1 No DNA
E
WT
Rab4A^Q72L
Rab4A^Q72L-KO
WT
Rab4A^Q72L
Rab4A^Q72L-KO
Actin (40 kD)
Cre (38 kD)
Rab4A (24 kD)
CD4+ T
CD8+ T

Figure S1. Generation of mice with constitutively active Rab4A^{Q72L} alleles. We replaced exons 3 of the Rab4A genomic lupus in 129SvJ-derived TC1 ES cells using a targeting vector with 5 kb upstream and 3 kB downstream genomic fragments harboring exon 2 and exons 4 and 5 respectively. Intron 3 contained a neomycin resistance cassette flanked by Frt recognition motifs. Intron 2 and intron 3 downstream of the neo cassette harbored LoxP sites to allow the removal of exon 3 by crossing with transgenic mice expressing Cre recombinase (panel A). The 129SvJ-derived TC1 ES cells heterozygous for the disrupted allele were microinjected into C57BL/6 blastocysts to generate chimeras. B) Chimeric males were mated with C57BL/6 females, and tail DNA of the offspring was tested for transmission of the targeted allele by PCR and sequencing of genomic DNA (panel C). Heterozygotes were back-crossed onto the C57Bl/6 line for ten generations. Heterozygotes were bred and wildtype (WT), heterozygote, and homozygote mice with floxed Rab4A^{Q72L} alleles were tracked by PCR genotyping (panel D). Homozygote Rab4A^{Q72L} were mated with heterozygote CD4^{Cre} transgenic mice to generate offspring lacking Rab4A expression in T cells (panel E). Expression of Rab4A was absent in CD4^{+} and CD8^{+} T cells of Rab4A^{Q72L}-KO mice. Cre was expressed CD4^{+} T cells of Rab4A^{Q72L}-KO mice. Cre was not expressed in peripheral CD8^{+} T cells as deletion of Rab4A had occurred at the developmental stage of double-positive T cells in the thymus, as originally described in this targeted deletion model [1].

Figure S2

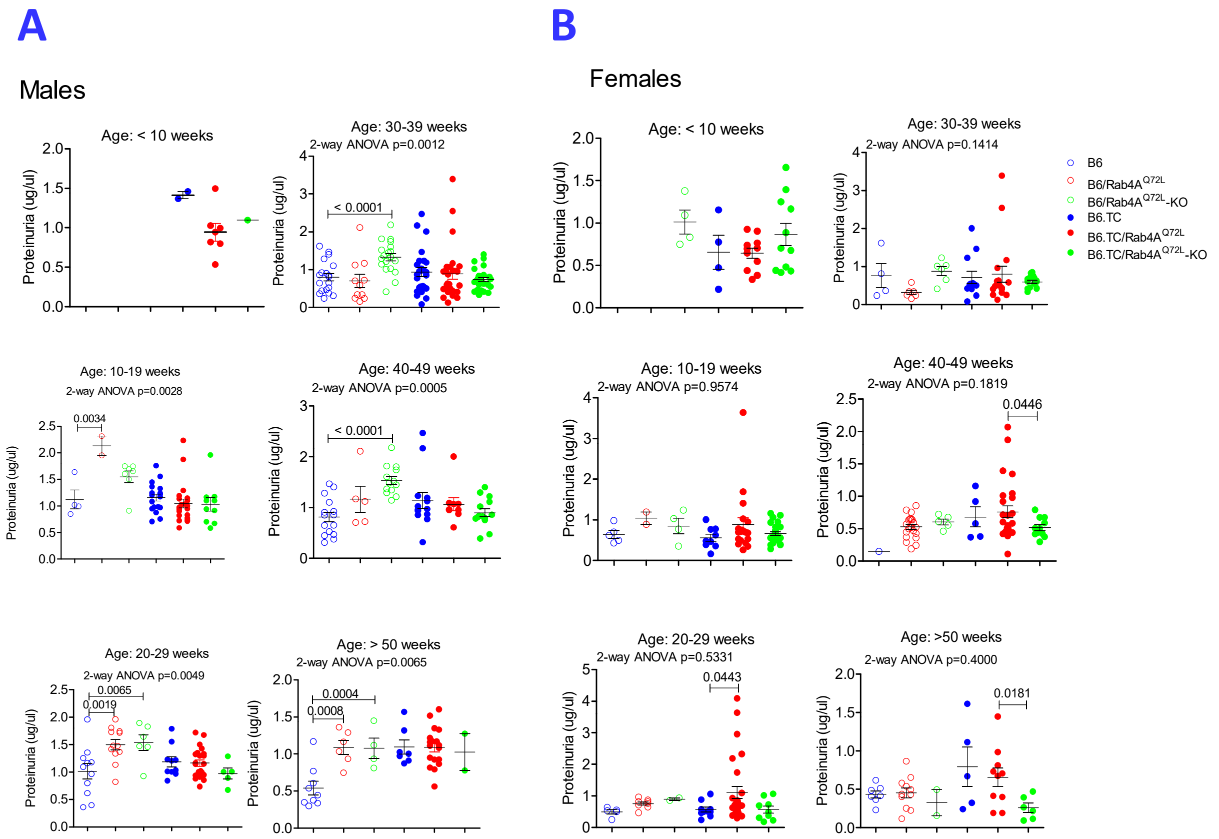

A
Males
Age: < 10 weeks
Age: 30-39 weeks
2-way ANOVA p=0.0012
< 0.0001
Age: 10-19 weeks
2-way ANOVA p=0.0028
0.0034
Age: 40-49 weeks
2-way ANOVA p=0.0005
< 0.0001
Age: 20-29 weeks
2-way ANOVA p=0.0049
0.0065
0.0019
Age: > 50 weeks
2-way ANOVA p=0.0065
0.0004
0.0008
Proteinuria (ug/ul)
B
Females
Age: < 10 weeks
Age: 30-39 weeks
2-way ANOVA p=0.1414
Age: 10-19 weeks
2-way ANOVA p=0.9574
Age: 40-49 weeks
2-way ANOVA p=0.1819
0.0446
Age: 20-29 weeks
2-way ANOVA p=0.5331
0.0443
Age: >50 weeks
2-way ANOVA p=0.4000
0.0181
Proteinuria (ug/ul)
B6
B6/Rab4A Q72L
B6/Rab4A Q72L-KO
B6.TC
B6.TC/Rab4A Q72L
B6.TC/Rab4A Q72L-KO

Figure S2. Effect of Rab4A on age-dependent changes in proteinuria in male and female B6 and B6.TC mice. A, Proteinuria was increased in young adult male (1.12±0.18 μg/μl) over female B6 mice at 10-19 weeks of age (0.65±0.96 μg/μl; p=0.0395). Proteinuria declined in males to 0.541 ug/ul by 50 weeks of age (p=0.0395). B, Proteinuria was reduced with remarkable consistency in B6.TC/Rab4a^{Q72L}-KO females relative to B6.TC/Rab4a^{Q72L} females at ages of 20-29, 30-39, 40-49, and >50 weeks. Overall two-way ANOVA p values are shown in the header of each figure panel, while Tukey's post-hoc test p values < 0.05 over brackets reflect comparison between experimental groups.

Figure S3

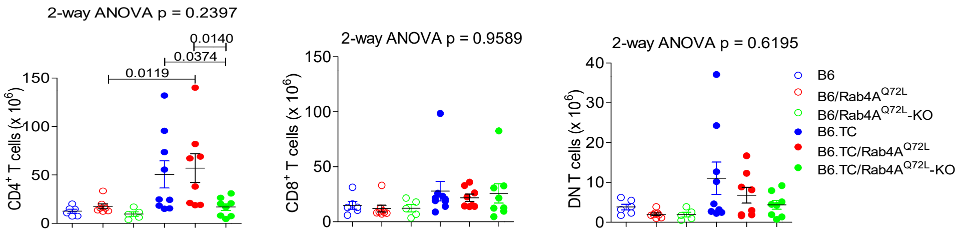
2-way ANOVA p = 0.2397
0.0140
0.0374
0.0119
CD4+ T cells (x 10^6)
150
100
50
0
2-way ANOVA p = 0.9589
CD8+ T cells (x 10^6)
150
100
50
0
2-way ANOVA p = 0.6195
DN T cells (x 10^6)
40
30
20
10
0
B6
B6/Rab4A Q72L
B6/Rab4A Q72L-KO
B6.TC
B6.TC/Rab4A Q72L
B6.TC/Rab4A Q72L-KO

Figure S3. Rab4A expands $CD4^+$ T cells at the expense of $CD8^+$ T cells both in B6 and B6.TC mice. Expansion of $CD4^+$ T cells by Rab4A in 20-week-old B6/Rab4A^{Q72L} and B6.TC/Rab4A^{Q72L} female mice relative to B6/Rab4A^{Q72L}-KO and B6.TC/Rab4A^{Q72L}-KO controls, respectively. Upper panel, representative flow cytometry dot plots; lower panel, cumulative analysis of absolute numbers of $CD4^+$ and $CD8^+$ and DN T cells. Overall two-way ANOVA p values are shown in the header of each figure panel, while Tukey's post-hoc test p values < 0.05 over brackets reflect comparison between experimental groups.

Figure S4

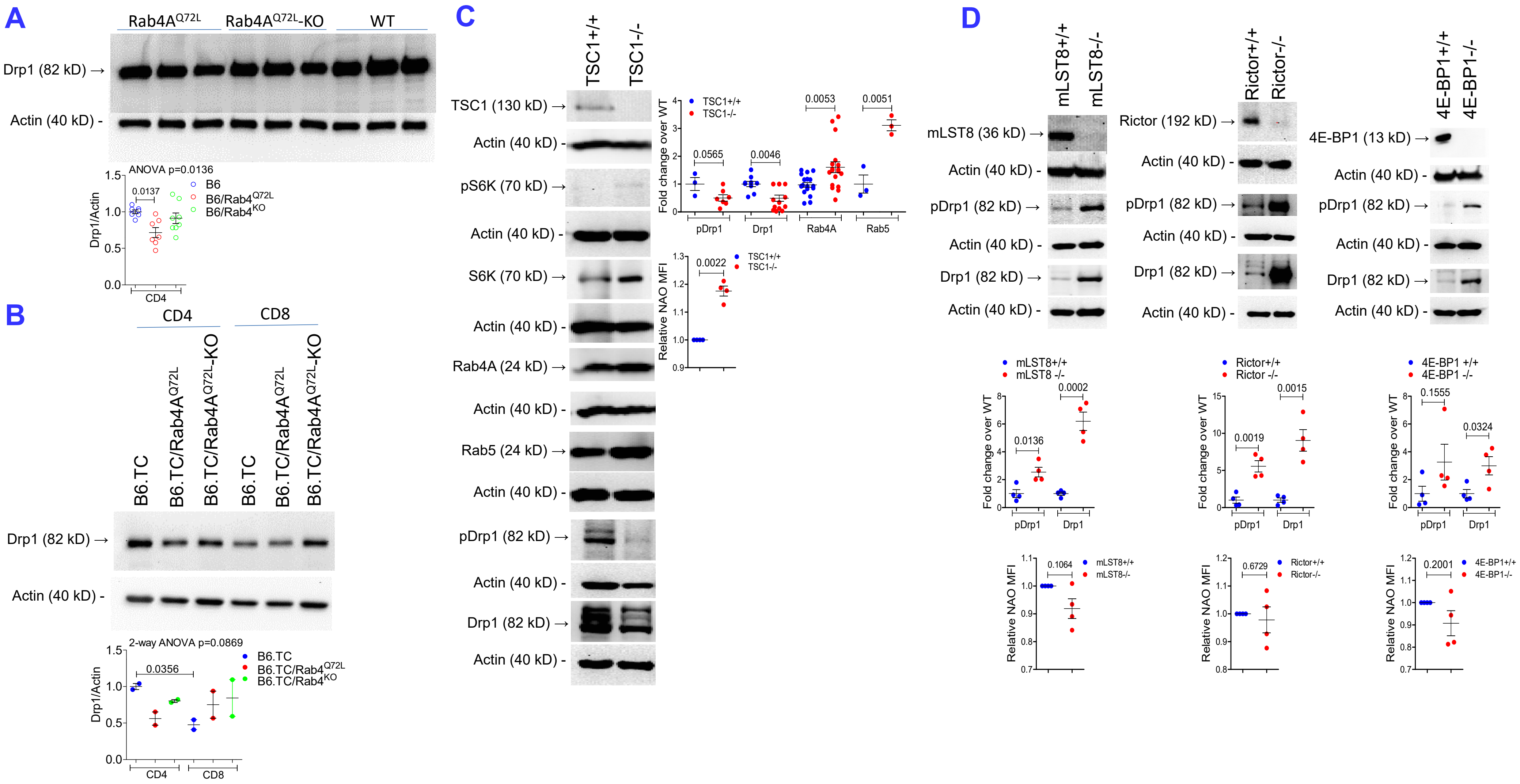

A
Rab4A^{Q72L}
Rab4A^{Q72L}-KO
WT
Drp1 (82 kD) →
Actin (40 kD) -
ANOVA p=0.0136
0.0137
B6
B6/Rab4^{Q72L}
B6/Rab4^{KO}
Drp1/Actin
CD4
B
CD4
CD8
B6.TC
B6.TC/Rab4A^{Q72L}
B6.TC/Rab4A^{Q72L}-KO
B6.TC
B6.TC/Rab4A^{Q72L}
B6.TC/Rab4A^{Q72L}-KO
Drp1 (82 kD) →
Actin (40 kD) -
2-way ANOVA p=0.0869
0.0356
B6.TC
B6.TC/Rab4^{Q72L}
B6.TC/Rab4^{KO}
Drp1/Actin
CD4
CD8
C
TSC1+/+
TSC1-/-
TSC1 (130 kD) →
Actin (40 kD) -
pS6K (70 kD) →
Actin (40 kD) -
S6K (70 kD) →
Actin (40 kD) -
Rab4A (24 kD) →
Actin (40 kD) -
Rab5 (24 kD) →
Actin (40 kD) -
pDrp1 (82 kD) →
Actin (40 kD) -
Drp1 (82 kD) →
Actin (40 kD) -
TSC1+/+
TSC1-/-
Fold change over WT
0.0565
0.0046
0.0053
0.0051
pDrp1
Drp1
Rab4A
Rab5
Relative NAO MFI
0.0022
TSC1+/+
TSC1-/-
D
mLST8+/+
mLST8-/-
mLST8 (36 kD) →
Actin (40 kD) -
pDrp1 (82 kD) →
Actin (40 kD) -
Drp1 (82 kD) →
Actin (40 kD) -
Rictor+/+
Rictor-/-
Rictor (192 kD) →
Actin (40 kD) -
pDrp1 (82 kD) →
Actin (40 kD) -
Drp1 (82 kD) →
Actin (40 kD) -
4E-BP1+/+
4E-BP1-/-
4E-BP1 (13 kD) →
Actin (40 kD) -
pDrp1 (82 kD) →
Actin (40 kD) -
Drp1 (82 kD) →
Actin (40 kD) -
mLST8+/+
mLST8 -/-
Fold change over WT
0.0136
0.0002
pDrp1
Drp1
Rictor+/+
Rictor -/-
Fold change over WT
0.0019
0.0015
pDrp1
Drp1
4E-BP1 +/+
4E-BP1 -/-
Fold change over WT
0.1555
0.0324
pDrp1
Drp1
Relative NAO MFI
0.1064
mLST8+/+
mLST8-/-
Relative NAO MFI
0.6729
Rictor+/+
Rictor-/-
Relative NAO MFI
0.2001
4E-BP1+/+
4E-BP1-/-

Figure S4. Effect of Rab4 and mTOR activation on Drp1 expression and mitochondrial accumulation in mouse CD4$^+$ and CD8$^+$ T cells and embryonic fibroblasts (MEFs). A, Western blot analysis of Drp1 expression in CD4$^+$ T cells of B6/WT, B6/Rab4A^{Q72L}, and B6/Rab4A^{Q72L}-KO mice. Representative western blots (top panel) and dot plot charts reflect mean ± SE of independent experiments (bottom panel); brackets reflect $p < 0.05$ indicating comparison to wild-type mice using two-tailed t-tests. Overall one-way ANOVA p values are shown in the header of each figure panel, while Sidak's post-hoc test p values < 0.05 over brackets reflect comparison between experimental groups. **B**, Western blot analysis of Drp1 expression in CD4$^+$ and CD8$^+$ T cells of B6.TC, B6.TC/Rab4A^{Q72L}, and B6.TC/Rab4A^{Q72L}-KO mice. Overall two-way ANOVA p values are shown in the header of each figure panel, while Tukey's post-hoc test p values < 0.05 over brackets reflect comparison between experimental groups. **C,** Western blot analysis of Rab4A, Drp1, and pDrp1 phosphorylated at serine 616 in TSC1-/- MEFs. Representative western blots (left panel) and bar charts reflect mean ± SE of 3 or more experiments (right panel); *, $p < 0.05$ indicates comparison to wild-type MEFs using two-tailed t-tests. **D,** Western blot analysis of Rab4A, Drp1, and pDrp1 phosphorylated at serine 616 in MEFs lacking mLST8 (mLST8-/-), Rictor (Rictor -/-), or 4E-BP1 (4E-BP1-/-). Representative western blots (left panel) and bar charts reflect mean ± SE of 3 or more experiments (right panel); *, $p < 0.05$ indicates comparison to wild-type MEFs using two-tailed t-tests.

Figure S5

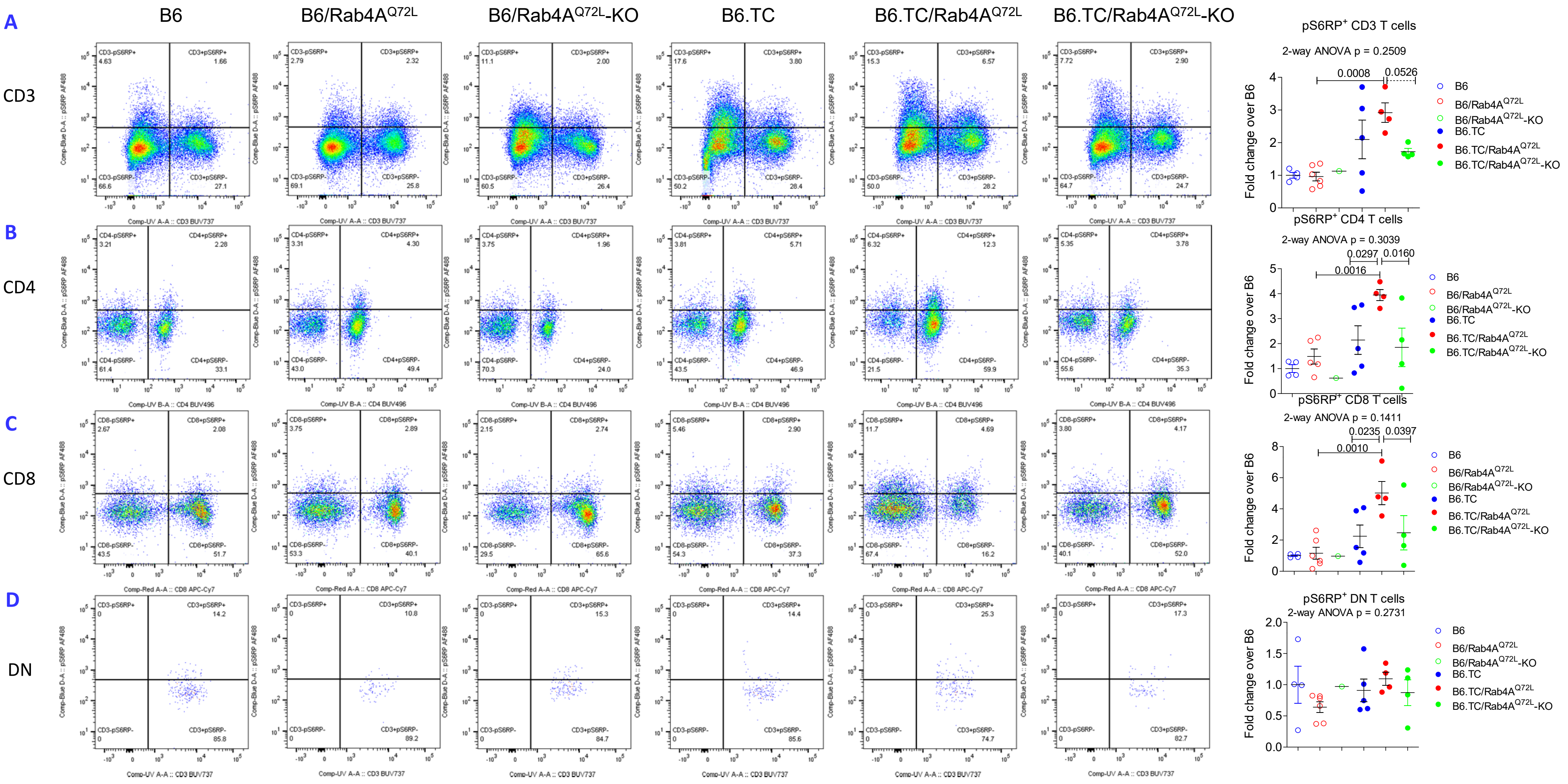

A
B
C
D
B6
B6/Rab4A^{Q72L}
B6/Rab4A^{Q72L}-KO
B6.TC
B6.TC/Rab4A^{Q72L}
B6.TC/Rab4A^{Q72L}-KO
CD3
CD4
CD8
DN
pS6RP^{+} CD3 T cells
2-way ANOVA p = 0.2509
0.0008
0.0526
pS6RP^{+} CD4 T cells
2-way ANOVA p = 0.3039
0.0297
0.0160
0.0016
pS6RP^{+} CD8 T cells
2-way ANOVA p = 0.1411
0.0235
0.0397
0.0010
pS6RP^{+} DN T cells
2-way ANOVA p = 0.2731
Fold change over B6

Figure S5. Activation of mTORC1 in CD3, CD4, CD8, and DN T cells is Rab4A-dependent in SLE. mTORC1 is activated in T cells of 20-week-old lupus-prone B6.TC/Rab4A^{Q72L} mice relative to B6/Rab4A^{Q72L} controls, which is reversed by the deletion of Rab4A in T cells of B6.TC/Rab4A^{Q72L}-KO mice. Consistent trends were observed in CD3 (**A**), CD4 (**B**), CD8 (**C**), and DN T cells (**D**), as shown by representative flow cytometry dot plots (left panels) and cumulative analysis (right panels). Overall two-way ANOVA p values are shown in the header of each figure panel, while Sidak's post-hoc test p values < 0.05 over brackets reflect comparison between experimental groups. Dash line reflects p value <0.1

Figure S6

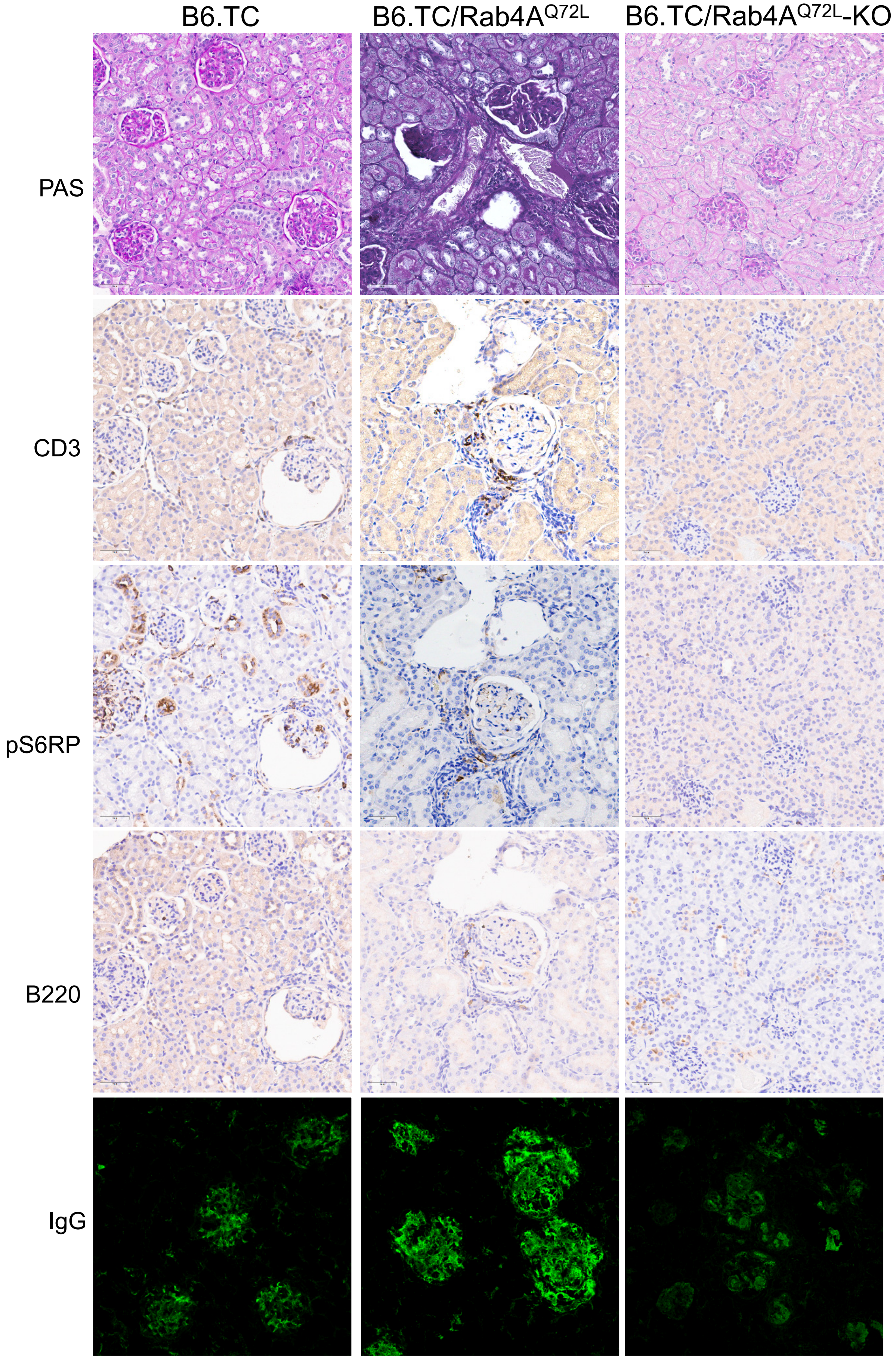
B6.TC
B6.TC/Rab4A^{Q72L}
B6.TC/Rab4A^{Q72L}-KO
PAS
CD3
pS6RP
B220
IgG

Figure S6. Detection CD3$^+$ T cells and B220$^+$ B cells, expression of pS6RP, and glomerular deposition of IgG and IgM in kidneys of 40-week-old female B6.TC, B6.TC/Rab4A^{Q72L} and B6.TC/Rab4A^{Q72L}-KO mice. Renal infiltration by CD3$^+$ T cells and B220$^+$ B cells and pS6RP were assessed by immunohistochemistry. Glomerular deposition of IgG and IgM was assessed by immunofluorescence microscopy. Scale bars are embedded into each microscopic image.

Figure S7

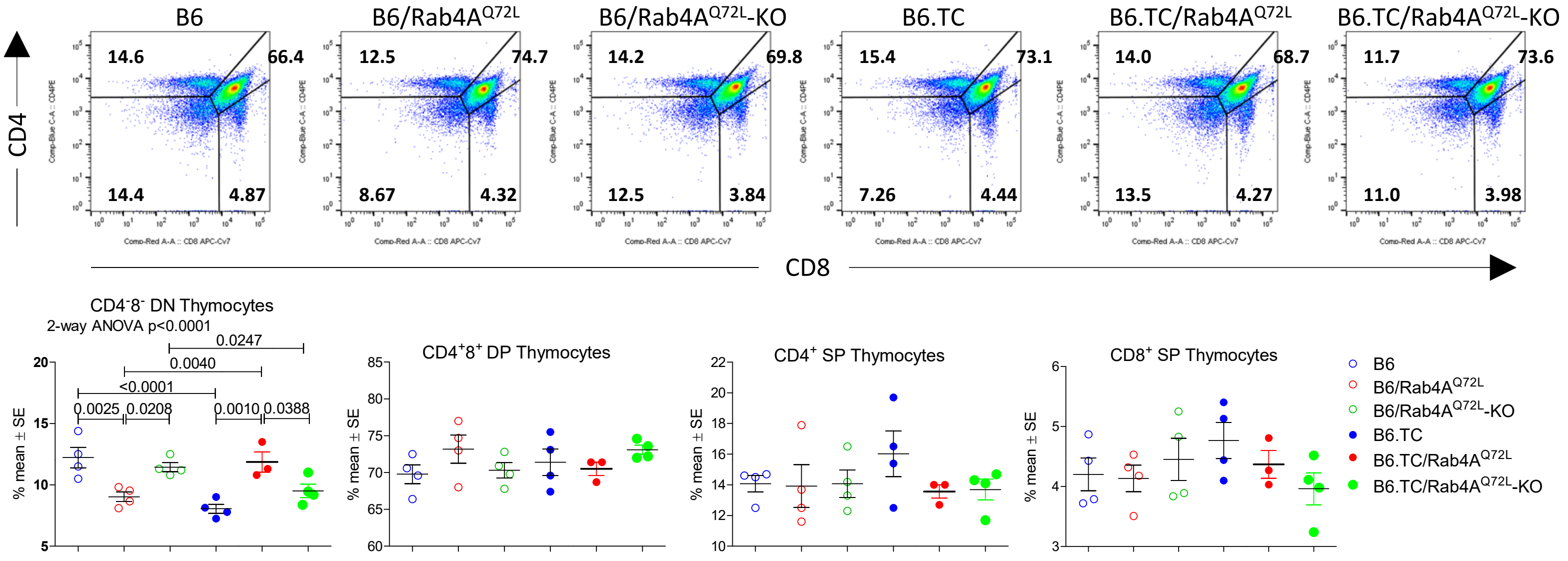
B6
B6/Rab4A^{Q72L}
B6/Rab4A^{Q72L}-KO
B6.TC
B6.TC/Rab4A^{Q72L}
B6.TC/Rab4A^{Q72L}-KO
14.6
66.4
14.4
4.87
12.5
74.7
8.67
4.32
14.2
69.8
12.5
3.84
15.4
73.1
7.26
4.44
14.0
68.7
13.5
4.27
11.7
73.6
11.0
3.98
CD4
CD8
CD4$^-$8$^-$ DN Thymocytes
2-way ANOVA p<0.0001
0.0247
0.0040
<0.0001
0.0025
0.0208
0.0010
0.0388
CD4$^+$8$^+$ DP Thymocytes
CD4$^+$ SP Thymocytes
CD8$^+$ SP Thymocytes
% mean ± SE
B6
B6/Rab4A^{Q72L}
B6/Rab4A^{Q72L}-KO
B6.TC
B6.TC/Rab4A^{Q72L}
B6.TC/Rab4A^{Q72L}-KO

Figure S7. Effect of Rab4A on T-cell development in the thymus. Abundance of $CD4^-CD8^-$ double-negative (DN) T cells, $CD4^+CD8^+$ double-positive (DP) T cells and $CD4^+$ or $CD8^+$ single-positive (SP) T cells were assessed in the thymus of 23 age-matched female mice carrying WT or constitutively active $Rab4^{Q72L}$ alleles, or lacking Rab4A in T cells in the B6 control and B6.TC lupus-prone backgrounds. These mice were of 25 ± 1.7 weeks of age when B6.TC/$Rab4A^{Q72L}$ females already show increased inflammation, elevated autoantibody production and proteinuria (**Figure 1**). Upper panel, representative flow cytometry dot plots. Lower panel, cumulative analysis show the mean ± SE of DN, DP, and SP T cells. Overall two-way ANOVA p values are shown in the header of each figure panel, while Sidak's post-hoc test p values < 0.05 over brackets reflect comparison between experimental groups.

Figure S8

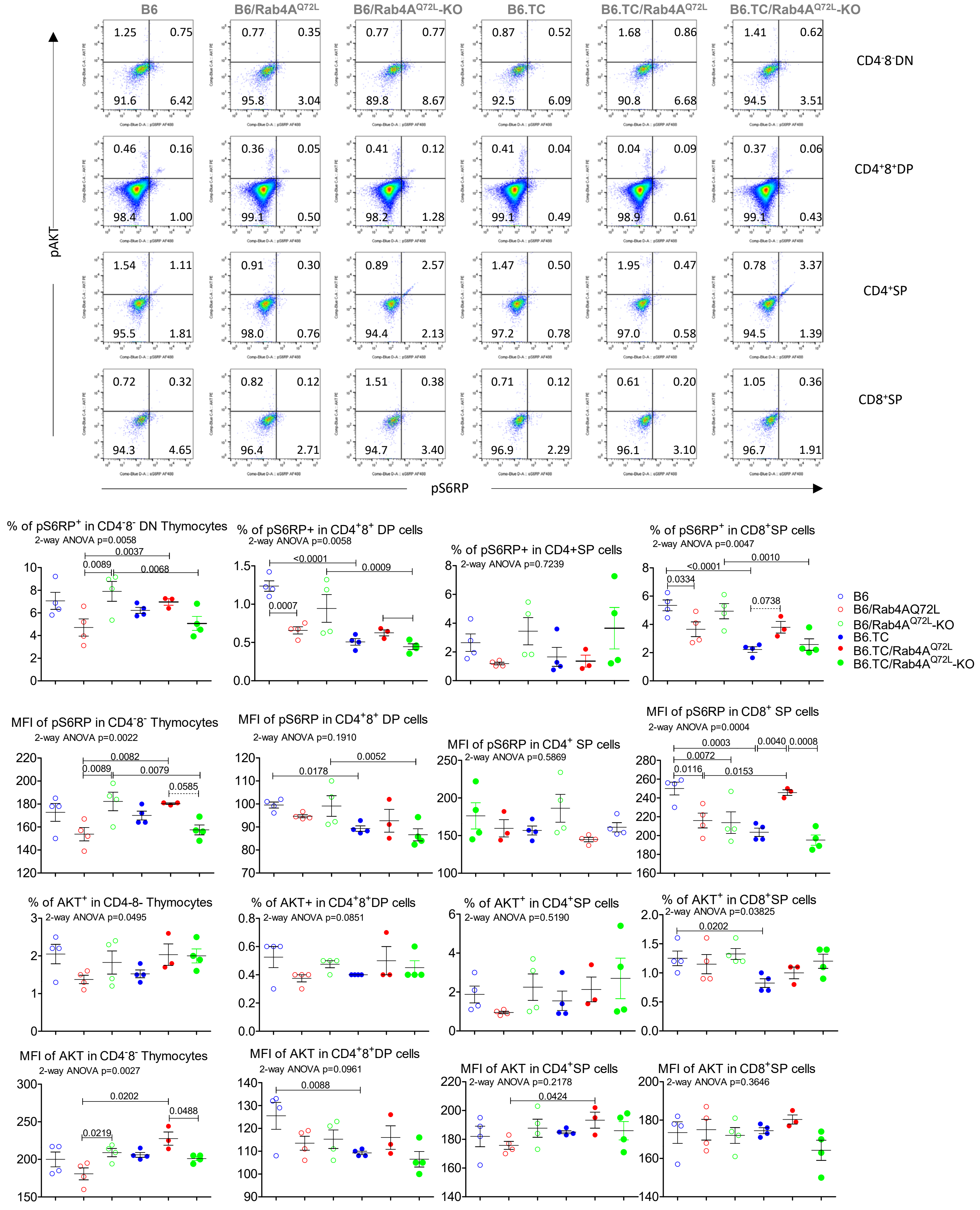

B6
B6/Rab4AQ72L
B6/Rab4AQ72L-KO
B6.TC
B6.TC/Rab4AQ72L
B6.TC/Rab4AQ72L-KO
CD4-8-DN
CD4+8+DP
CD4+SP
CD8+SP
pAKT
pS6RP
% of pS6RP+ in CD4-8- DN Thymocytes
2-way ANOVA p=0.0058
% of pS6RP+ in CD4+8+ DP cells
2-way ANOVA p=0.0058
% of pS6RP+ in CD4+SP cells
2-way ANOVA p=0.7239
% of pS6RP+ in CD8+SP cells
2-way ANOVA p=0.0047
MFI of pS6RP in CD4-8- Thymocytes
2-way ANOVA p=0.0022
MFI of pS6RP in CD4+8+ DP cells
2-way ANOVA p=0.1910
MFI of pS6RP in CD4+ SP cells
2-way ANOVA p=0.5869
MFI of pS6RP in CD8+ SP cells
2-way ANOVA p=0.0004
% of AKT+ in CD4-8- Thymocytes
2-way ANOVA p=0.0495
% of AKT+ in CD4+8+DP cells
2-way ANOVA p=0.0851
% of AKT+ in CD4+SP cells
2-way ANOVA p=0.5190
% of AKT+ in CD8+SP cells
2-way ANOVA p=0.03825
MFI of AKT in CD4-8- Thymocytes
2-way ANOVA p=0.0027
MFI of AKT in CD4+8+DP cells
2-way ANOVA p=0.0961
MFI of AKT in CD4+SP cells
2-way ANOVA p=0.2178
MFI of AKT in CD8+SP cells
2-way ANOVA p=0.3646

Figure S8. Effect of Rab4A on activation of mTORC1 and mTORC2 in T cells of the thymus. Activation of mTORC1 and mTORC2 was assessed within of $CD4^{-}CD8^{-}$ double-negative (DN) T cells, $CD4^{+}CD8^{+}$ double-positive (DP) T cells and $CD4^{+}$ or $CD8^{+}$ single-positive (SP) T cells of the thymus from 23 age-matched female mice carrying WT or constitutively active $Rab4^{Q72L}$ alleles, or lacking Rab4A in T cells in the B6 control and B6.TC lupus-prone backgrounds, as described in **Figure S5**. Upper panel, representative flow cytometry dot plots. Lower panel, cumulative analysis show the mean ± SE of DN, DP, and SP T cells. Overall two-way ANOVA p values are shown in the header of each figure panel, while Sidak's post-hoc test p values < 0.05 over brackets reflect comparison between experimental groups.

Figure S9

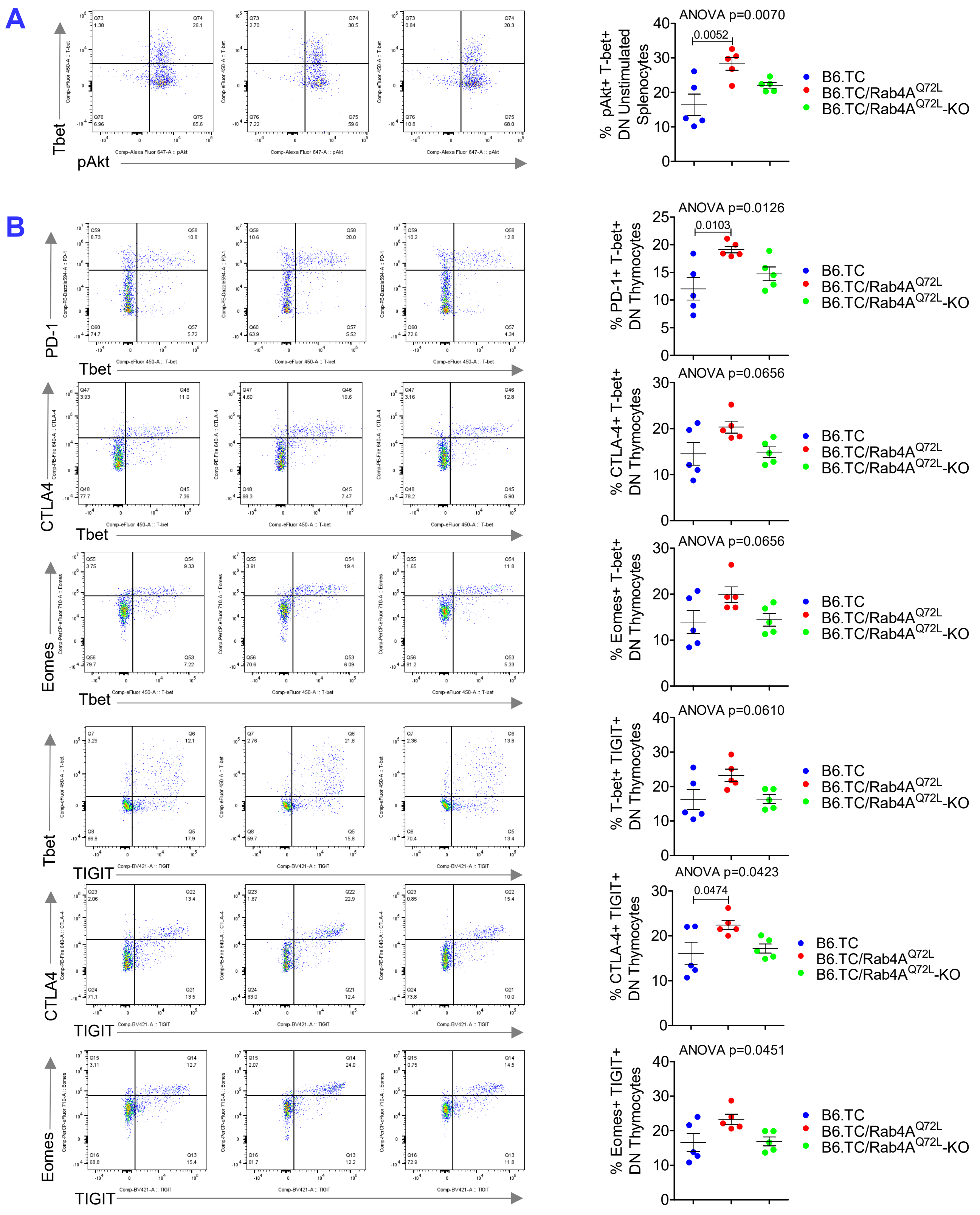

A
Tbet
pAkt
ANOVA p=0.0070
0.0052
% pAkt+ T-bet+ DN Unstimulated Splenocytes
B6.TC
B6.TC/Rab4A^{Q72L}
B6.TC/Rab4A^{Q72L}-KO
B
PD-1
Tbet
ANOVA p=0.0126
0.0103
% PD-1+ T-bet+ DN Thymocytes
CTLA4
Tbet
ANOVA p=0.0656
% CTLA-4+ T-bet+ DN Thymocytes
Eomes
Tbet
ANOVA p=0.0656
% Eomes+ T-bet+ DN Thymocytes
Tbet
TIGIT
ANOVA p=0.0610
% T-bet+ TIGIT+ DN Thymocytes
CTLA4
TIGIT
ANOVA p=0.0423
0.0474
% CTLA-4+ TIGIT+ DN Thymocytes
Eomes
TIGIT
ANOVA p=0.0451
% Eomes+ TIGIT+ DN Thymocytes

Figure S9. Effect of Rab4A on markers of exhaustion in T cells of B6.TC, B6.TC/Rab4A^{Q72L}, and B6.TC/Rab4A^{Q72L} -KO mice. Markers of exhaustion, T-bet, Eomes, TIGIT, PD-1, PL-L2, and CTLA-4 [2] were examine in CD4^{+}, CD8^{+}, and DN T cells from the spleen and DN, DP, CD4^{+}, and CD8^{+} SP T cells from the thymus of B6.TC, B6.TC/Rab4A^{Q72L}, and B6.TC/Rab4A^{Q72L} -KO mice, using five age-matched females per genotype.These mice were of 38 ± 0.6 weeks of age when B6.TC/Rab4A^{Q72L} females already show increased inflammation, elevated autoantibody production and proteinuria. **A**, Expansion of T-bet^{+}/pAkt^{+} DN T cells in the spleen of B6.TC/Rab4A^{Q72L} mice. Left panel, representative flow cytometry dot plots. Right panel, cumulative analysis shows the mean ± SE of DN T cells; p values represent comparison with two-tailed unpaired t-test. **B**, Expansion of PD-1^{+}T-bet^{+}, CTLA4^{+}T-bet^{+}, Eomes^{+}T-bet^{+}, TIGIT^{+}T-bet^{+}, TIGIT^{+}CTLA4^{+}, and TIGIT^{+}Eomes^{+} DN T cells in the thymus of B6.TC/Rab4A^{Q72L} mice over those in B6.TC/Rab4A^{Q72L}-KO mice. Left panel, representative flow cytometry dot plots. Right panel, cumulative analyses show the mean ± SE of DN T cells. Overall one-way ANOVA p values are shown in the header of each figure panel, while Tukey's post-hoc test p values < 0.05 over brackets reflect comparison between experimental groups.

Figure S10

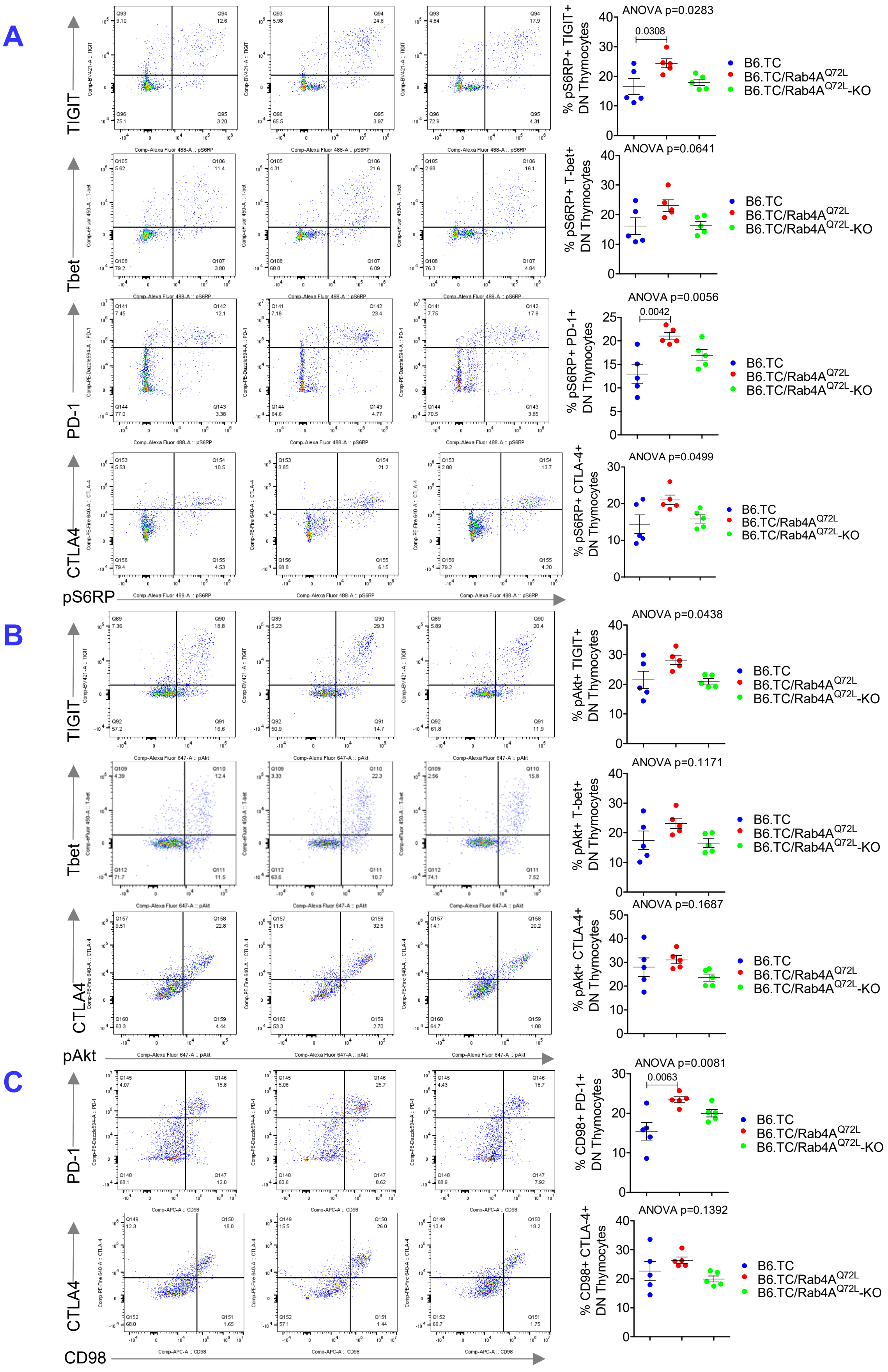

A
TIGIT
Tbet
PD-1
CTLA4
pS6RP
ANOVA p=0.0283
0.0308
% pS6RP+ TIGIT+ DN Thymocytes
ANOVA p=0.0641
% pS6RP+ T-bet+ DN Thymocytes
ANOVA p=0.0056
0.0042
% pS6RP+ PD-1+ DN Thymocytes
ANOVA p=0.0499
% pS6RP+ CTLA-4+ DN Thymocytes
B
TIGIT
Tbet
CTLA4
pAkt
ANOVA p=0.0438
% pAkt+ TIGIT+ DN Thymocytes
ANOVA p=0.1171
% pAkt+ T-bet+ DN Thymocytes
ANOVA p=0.1687
% pAkt+ CTLA-4+ DN Thymocytes
C
PD-1
CTLA4
CD98
ANOVA p=0.0081
0.0063
% CD98+ PD-1+ DN Thymocytes
ANOVA p=0.1392
% CD98+ CTLA-4+ DN Thymocytes
B6.TC
B6.TC/Rab4A^{Q72L}
B6.TC/Rab4A^{Q72L}-KO

Figure S10. Coordinate impact by Rab4A on markers of exhaustion, activation of mTORC1 and mTORC2, and expression of CD98 in DN T cells of B6.TC, B6.TC/Rab4A^{Q72L}, and B6.TC/Rab4A^{Q72L} -KO mice. A, Markers of exhaustion, T-bet, Eomes, TIGIT, PD-1, PL-L2, and CTLA-4 [2] were examined relative to activation of mTORC1 in DN, DP, CD4^{+}, and CD8^{+} SP T cells from the thymus of B6.TC, B6.TC/Rab4A^{Q72L}, and B6.TC/Rab4A^{Q72L} -KO mice, using five age-matched females per genotype. These mice were of 38 ± 0.6 weeks of age when B6.TC/Rab4A^{Q72L} females already show increased inflammation, elevated autoantibody production and proteinuria (**Figure S2**). Left panel, representative flow cytometry dot plots of DN T cells. Right panel, cumulative analyses show the mean ± SE of DN T cells. Overall one-way ANOVA p values are shown in the header of each figure panel, while Tukey's post-hoc test p values < 0.05 over brackets reflect comparison between experimental groups. **B,** Markers of exhaustion, T-bet, Eomes, TIGIT, PD-1, PL-L2, and CTLA-4 [2] were examined relative to activation of mTORC2 activation in DN, DP, CD4^{+}, and CD8^{+} SP T cells from the thymus of B6.TC, B6.TC/Rab4A^{Q72L}, and B6.TC/Rab4A^{Q72L} -KO mice, using five age-matched females per genotype. Left panel, representative flow cytometry dot plots of DN T cells. Right panel, cumulative analyses show the mean ± SE of DN T cells. Overall one-way ANOVA p values are shown in the header of each figure panel, while Tukey's post-hoc test p values $<$ 0.05 over brackets reflect comparison between experimental groups. **C,** Markers of exhaustion, T-bet, Eomes, TIGIT, PD-1, PL-L2, and CTLA-4 [2] were examined relative to CD98 expression in DN, DP, CD4^{+}, and CD8^{+} SP T cells from the thymus of B6.TC, B6.TC/Rab4A^{Q72L}, and B6.TC/Rab4A^{Q72L} -KO mice, using five age-matched females per genotype. Left panel, representative flow cytometry dot plots of DN T cells. Right panel, cumulative analyses show the mean ± SE of DN T cells. Overall one-way ANOVA p values are shown in the header of each figure panel, while Tukey's post-hoc test p values < 0.05 over brackets reflect comparison between experimental groups.

Figure S11

A

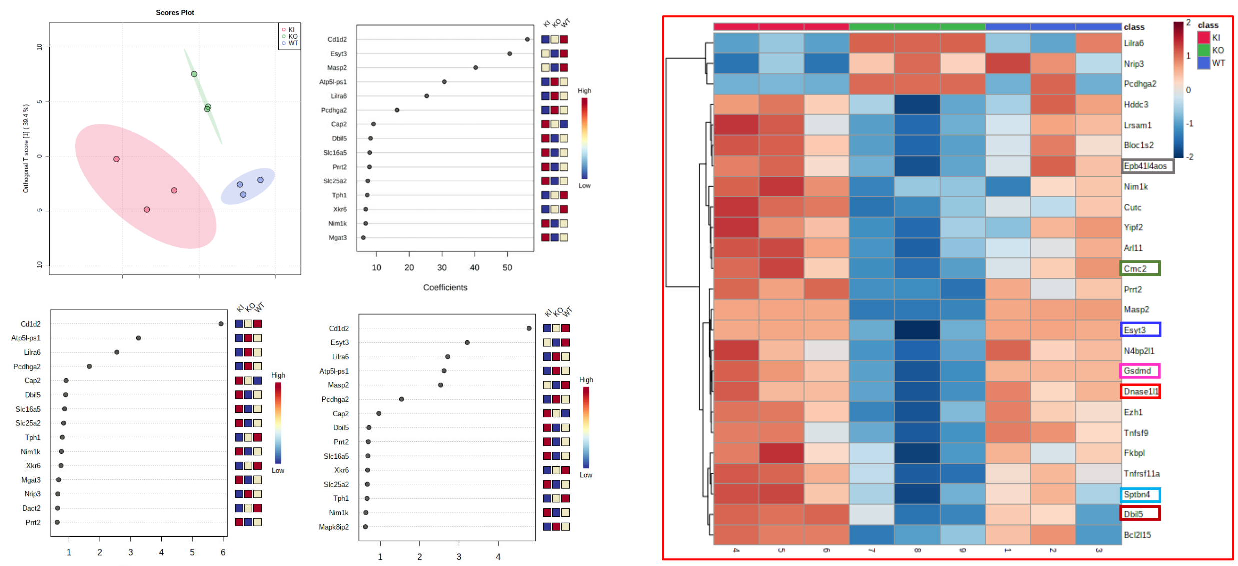

B

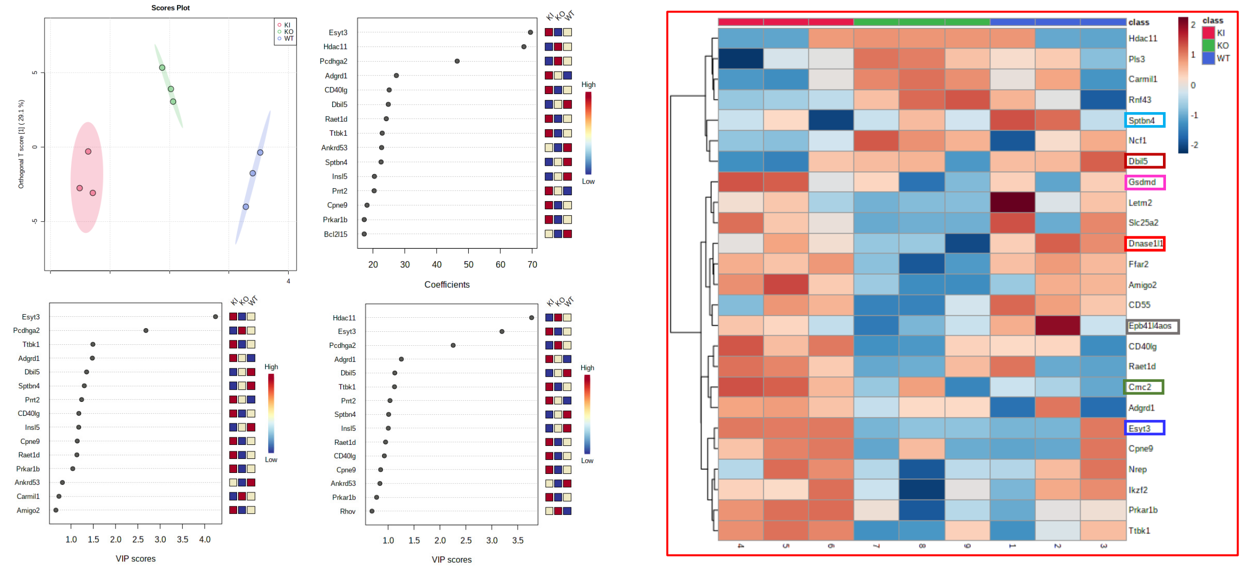

C

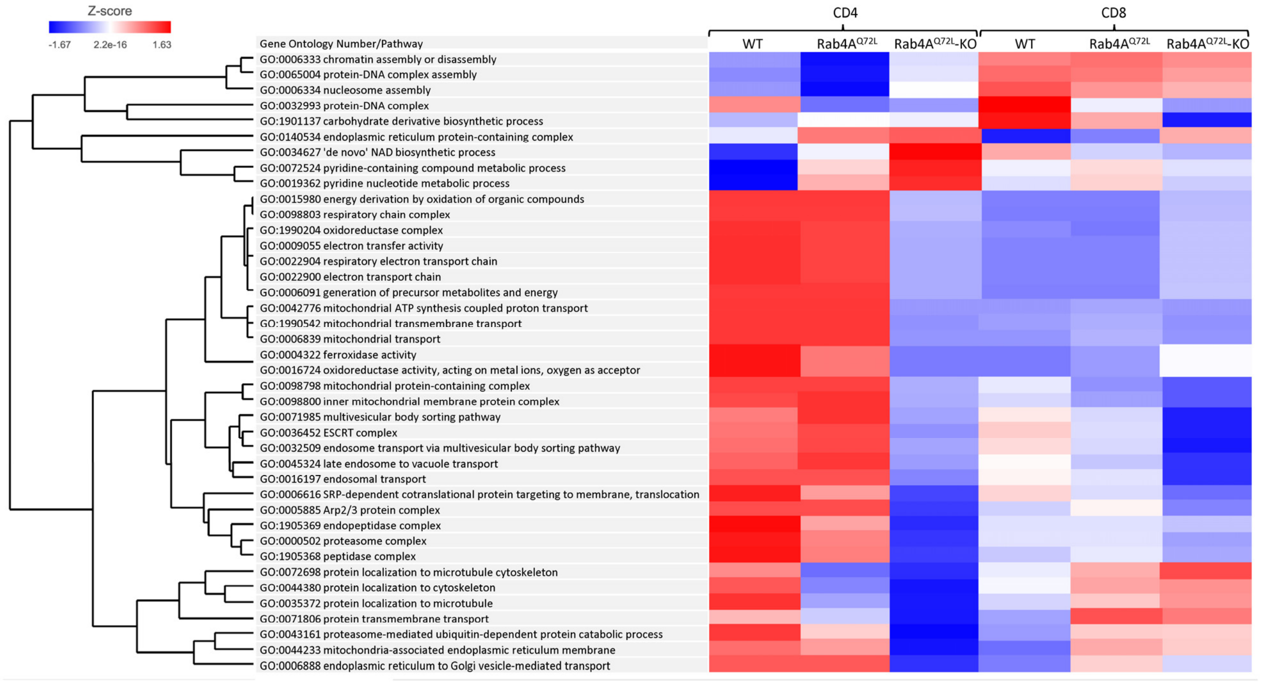

Figure S11. Gene expression analysis by RNAseq revealed distortion of mitochondrial and endosome traffic pathways by Rab4A into opposite directions between $CD4^+$ T cells and $CD8^+$ T cells. A, Discrimination of gene expression amongst $CD4^+$ T cells from B6.TC (WT), B6.TC/Rab4A^{Q72L}(KI) and B6.TC/Rab4A^{Q72L}-KO (KO) mice by orthogonal PLSDA analysis. Individual genes driving discrimination of two components are shown by VIP scores. Coefficient scores show 15 most impactful genes. Heat diagrams show expression of 25 genes providing discrimination by ANOVA at false discovery rate (FDR) p <0.05. **B,** Discrimination of gene expression amongst $CD8^+$ T cells from B6.TC (WT), B6.TC/Rab4A^{Q72L} (KI) and B6.TC/Rab4A^{Q72L}-KO (KO) mice by orthogonal PLSDA analysis. Individual genes driving discrimination of two components are shown by VIP scores. Coefficient scores show 15 most impactful genes. Heat diagrams show expression of 25 genes providing discrimination by ANOVA at FDR p <0.05. Genes affected by Rab4A both in $CD4^+$ and $CD8^+$ T cells are visualized by brackets with matching colors. **C,** Pathway analysis of gene expression in $CD4^+$ T cells and $CD8^+$ T cells from B6.TC (WT), B6.TC/Rab4A^{Q72L} (KI) and B6.TC/Rab4A^{Q72L}-KO (KO) mice. Heat diagrams show the top 40 of 12,777 pathways affected by Rab4A at FDR p<0.05 across all samples analyzed using the Partek Genomics Suite analysis software. Pathway annotations can be optimally read at 200% zoom.

Figure S12

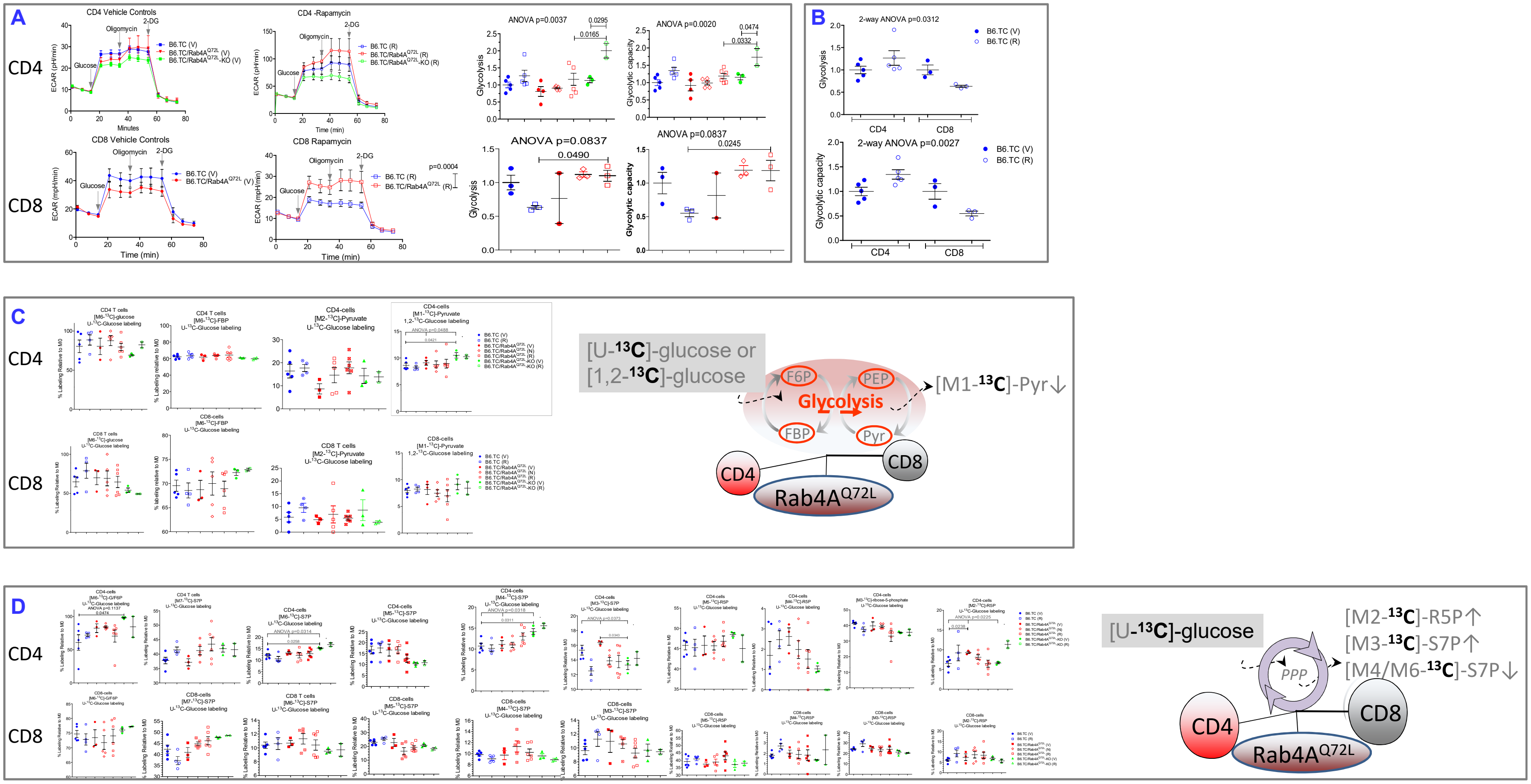
A
CD4
CD8
CD4 Vehicle Controls
CD4 -Rapamycin
CD8 Vehicle Controls
CD8 Rapamycin
Glucose
Oligomycin
2-DG
ECAR (pH/min)
ECAR (mpH/min)
Time (min)
Minutes
B6.TC (V)
B6.TC (R)
p=0.0004
ANOVA p=0.0037
ANOVA p=0.0020
ANOVA p=0.0837
0.0295
0.0165
0.0474
0.0332
0.0490
0.0245
Glycolysis
Glycolytic capacity
B
2-way ANOVA p=0.0312
2-way ANOVA p=0.0027
B6.TC (V)
B6.TC (R)
C
[U-13C]-glucose or
[1,2-13C]-glucose
F6P
PEP
FBP
Pyr
Glycolysis
[M1-13C]-Pyr↓
CD4
CD8
Rab4AQ72L
D
[U-13C]-glucose
PPP
[M2-13C]-R5P↑
[M3-13C]-S7P↑
[M4/M6-13C]-S7P↓

Figure S12. The Rab4A-mTOR axis restricts metabolic flux through glycolysis and the PPP in CD4$^+$ T cells. The impact of Rab4A activation and treatment with rapamycin and NAC on glucose metabolism in CD4$^+$ and CD8$^+$ T cells were examined following stimulation through CD3 and CD28 for 72 hours. **A,** Measurement of extracellular acidification rate (ECAR, mpH/min) in CD4$^+$ and CD8$^+$ T cells by using a Seahorse Metabolic Analyzer. ECAR levels of B6.TC, B6.TC/Rab4A^{Q72L} and B6.TC/Rab4A^{Q72L}-KO mice were compared in mice treated with 0.2% CMC (Vehicle panel) or rapamycin dissolved in 0.2% CMC (Rapamycin panel). Dot plot charts reflect mean ± SE of cumulative analyses of glycolysis and glycolytic capacity. Overall one-way ANOVA p values are shown in the header of each figure panel, while Sidak's post-hoc test p values < 0.05 over brackets reflect comparison between experimental groups. Due to limited availability of cells, the scope of studies was constrained in CD8 T cells. **B,** Opposing effects of rapamycin on glycolysis in CD4$^+$ and CD8$^+$ T cells of B6.TC mice. Statistical analyses were done by two-way ANOVA. **C,** Assessment of metabolic flux through glycolysis in CD4$^+$ and CD8$^+$ T cells stimulated via CD3 and CD28 for 72 hours and labelled with [U-^{13}C]-glucose or [1,2-^{13}C]-glucose stable isotope tracer for analysis by high-resolution LC-MS/MS. Overall one-way ANOVA p values are shown in the header of each figure panel, while Tukey's post-hoc test p values < 0.05 over brackets reflect comparison between experimental groups. **D,** Assessment of [U-^{13}C]-glucose flux through the PPP in CD4$^+$ and CD8$^+$ T cells. Overall one-way ANOVA p values are shown in the header of each figure panel, while Tukey's post-hoc test p values < 0.05 over brackets reflect comparison between experimental groups.

Figure S13

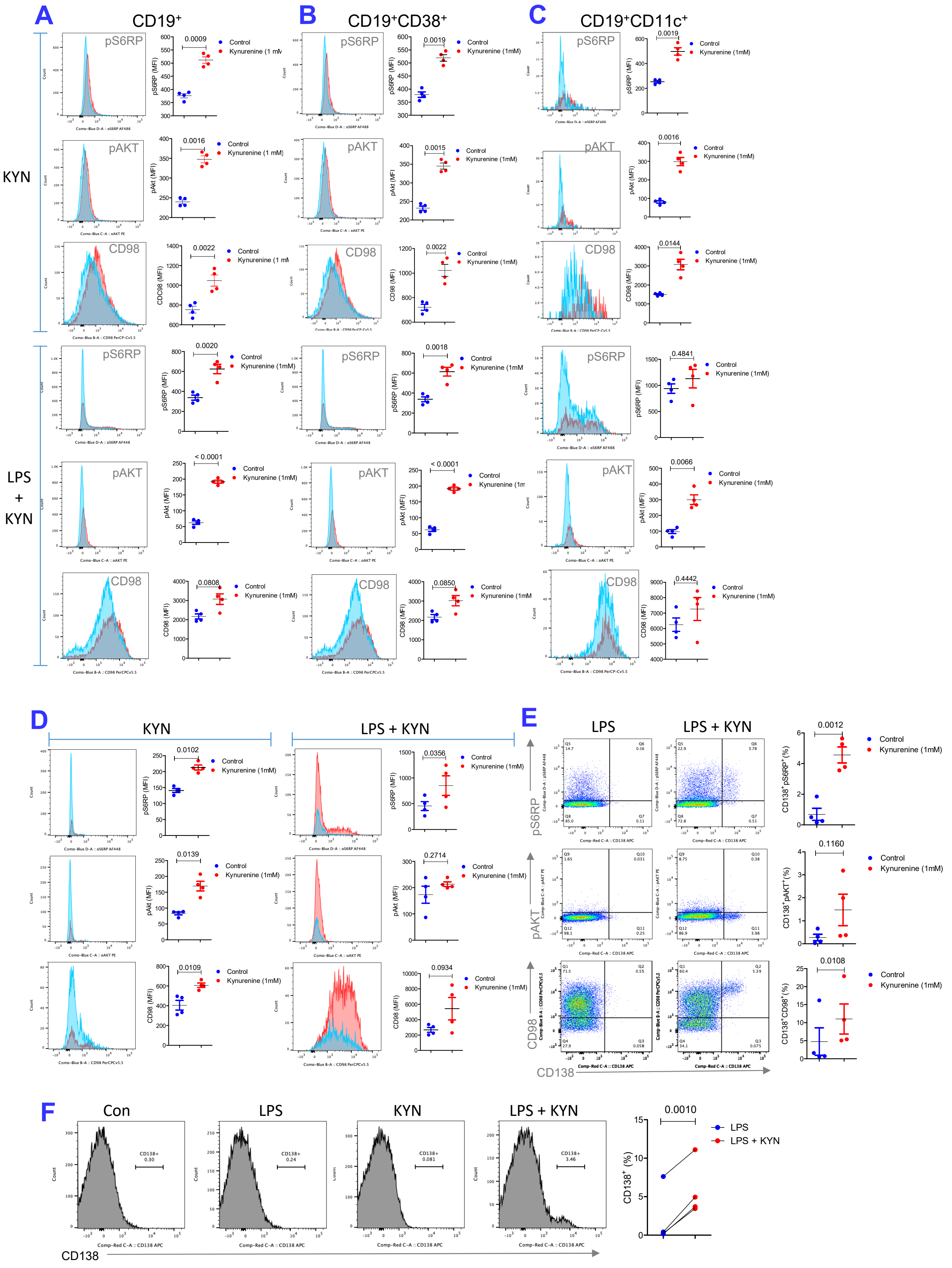

A
CD19+
B
CD19+CD38+
C
CD19+CD11c+
KYN
LPS + KYN
D
KYN
LPS + KYN
E
LPS
LPS + KYN
F
Con
LPS
KYN
LPS + KYN
CD138
Control
Kynurenine (1mM)

Figure S13. **Effect of kynurenine (KYN) on activation of mTORC1 and mTORC2 and expression of CD98 in CD19$^+$ B cells.** Splenocytes from female B6 mice were stimulated with 1 mM kynurenine alone or together with CD3/CD28 or LPS for 72 hours in vitro, as indicated for each panel. Following staining for surface expression of CD3, CD4, CD8, CD11c, CD19, CD38, CD138, and CD98, cell were fixed and permeabilized and activities of mTORC1 and mTORC2 were measured by intracellular staining for pS6RP and pAkt, respectively.

A, Effect of KYN with and without concurrent LPS stimulation on mTORC1 and mTORC2 activities and CD98 expression in CD19$^+$ B cells. Representative flow cytometry histograms and Graphpad charts with mean ± SE of four independent experiments are shown. Brackets represent p values < 0.05 by comparison using two-tailed paired t-test. **B**, Effect of KYN with and without concurrent LPS stimulation on mTORC1 and mTORC2 activities and CD98 expression in CD19$^+$CD38$^+$ B cells. Representative flow cytometry histograms and Graphpad charts with mean ± SE of four independent experiments are shown. Brackets represent p values < 0.05 by comparison using two-tailed paired t-test. **C**, Effect of KYN with and without concurrent LPS stimulation on mTORC1 and mTORC2 activities and CD98 expression in CD19$^+$ CD11c$^+$ B cells. Representative flow cytometry histograms and Graphpad charts with mean ± SE of four independent experiments are shown. Brackets represent p values < 0.05 by comparison using two-tailed paired t-test. **D**, Effect of KYN with and without concurrent LPS stimulation on mTORC1 and mTORC2 activities and CD98 expression in CD138$^+$ plasma cells. Representative flow cytometry histograms and Graphpad chars with mean ± SE of four independent experiments are shown. Brackets represent p values < 0.05 by comparison using two-tailed paired t-test. **E**, Effect of KYN with and without concurrent LPS stimulation on mTORC1 and mTORC2 activities relative to CD138$^+$ expression. Representative flow cytometry dot plots and Graphpad charts with mean ± SE of four independent experiments are shown. Brackets represent p values $<$ 0.05 by comparison using two-tailed paired t-test. **F**, Effect of KYN with and without concurrent

LPS stimulation on prevalence of $CD138^+$ plasma cells. Representative flow cytometry histograms and Graphpad charts with mean ± SE of four independent experiments are shown. Brackets represent p values < 0.05 by comparison using two-tailed paired t-test.

Figure S14

6/28/2022 HeLa CD98 knockdown

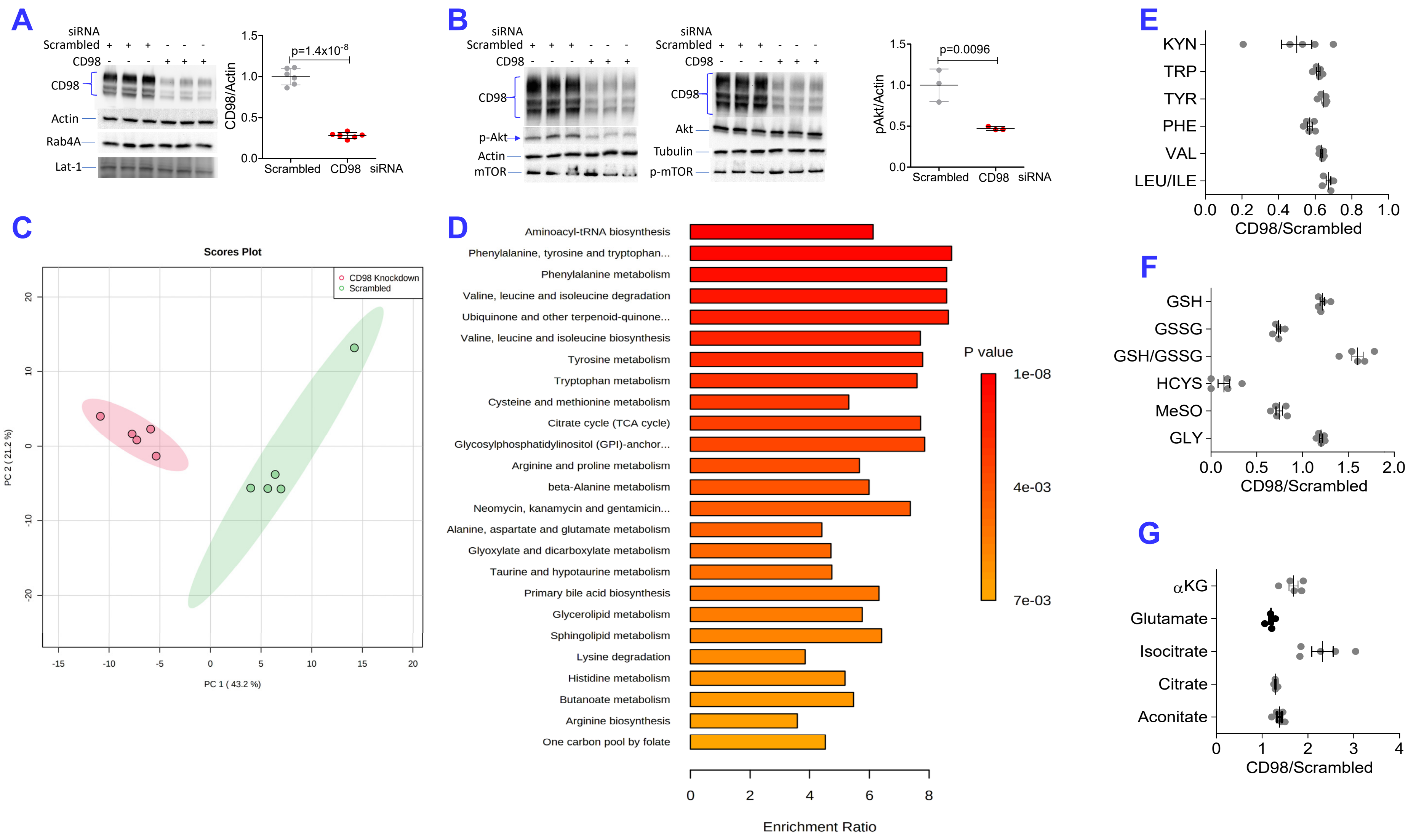

Figure S14. **CD98 expression controls intracellular levels of branched and aromatic amino acids and kynurenine.** **A**, Knockdown of CD98 expression by siRNA in HeLa cells. Left panel indicates representative western blot detection of CD98 and LAT1 protein levels relative to β-actin. Right panel indicates cumulative analysis of 5 replicate experiments, showing knockdown of CD98. $p<0.05$ represents unpaired two-tailed t-test. **B**, Left panel, representative western blot detection of pAkt and mTOR protein levels upon CD98 knockdown; Middle panel, representative western blot detection of Akt and p-mTOR protein levels upon CD98 knockdown; Right panel, cumulative analysis of pAkt/actin levels upon siRNA-mediated knockdown of CD98. $p<0.05$ represents unpaired two-tailed t-test. **C**, Principal component analysis of the effect of CD98 knockdown on the metabolome using Metaboanalyst 5.0, **D**, Effect of CD98 knock down of metabolic pathways. 25 of 84 KEGG pathways affected at FDR $p < 0.05$ are displayed. **E**, Effect of siRNA-mediated CD98 knockdown on intracellular levels of branched chain (VAL, LEU/ILE) and aromatic amino acids (PHE, TYR, TRP) and KYN levels relative to scrambled siRNA control. Changes are all significant at $p<0.05$ using unpaired two-tailed t-test. **F**, Effect of CD98 knockdown on intracellular levels of GSH, GSSG, GSH/GSSG ratio, methionine sulfoxide (MeSO), and homocysteine (HCYS). Data represent the effect of CD98 siRNA relative to scrambled siRNA control in 5 replicate experiments. Changes are all significant at $p<0.05$ using unpaired two-tailed t-test. **G**, Effect of CD98 knockdown on intracellular levels of TCA metabolites, αKG, glutamate, citrate, isocitrate, aconitate. Data represent the effect of CD98 siRNA relative to scrambled siRNA control in 5 replicate experiments. Changes are significant at $p<0.05$ using unpaired two-tailed t-test.

Figure S15

A

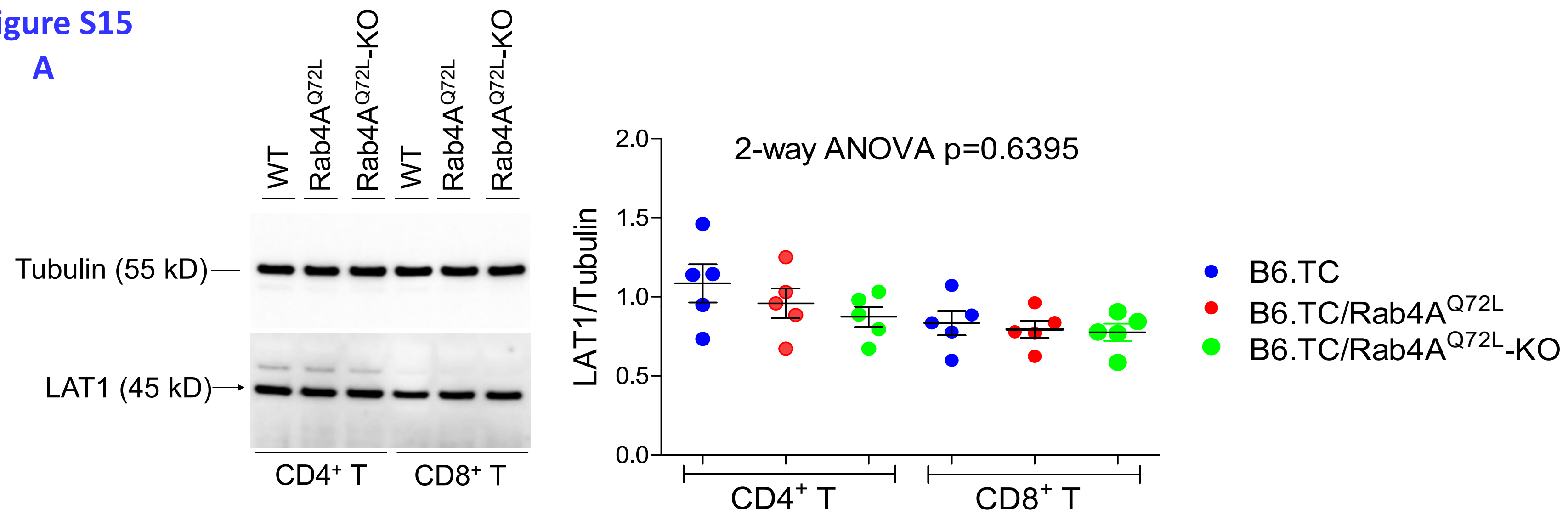

B

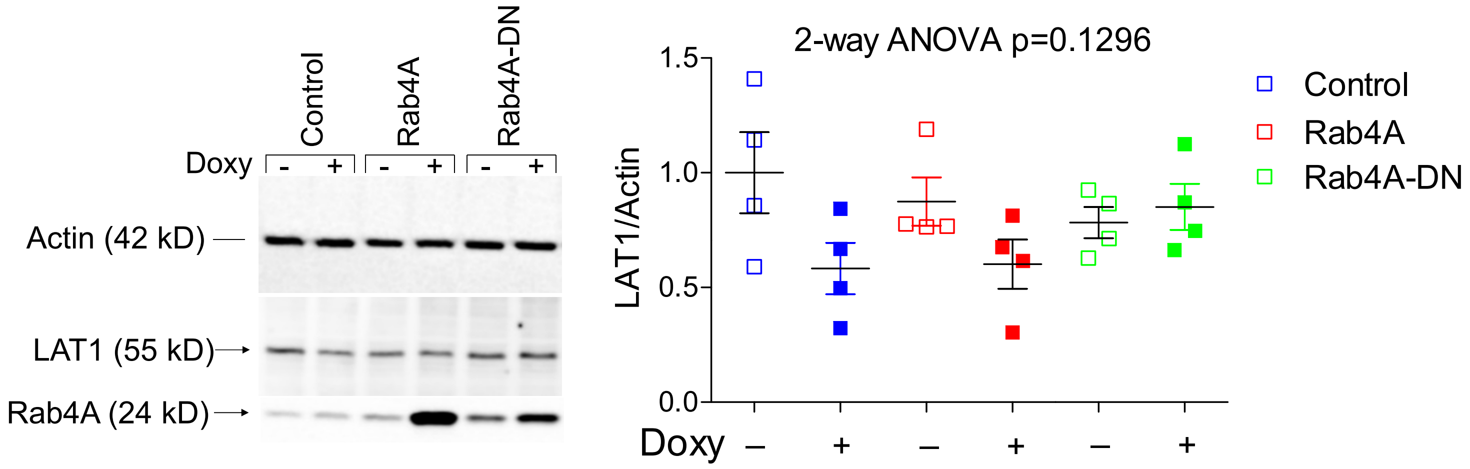

Figure S15. **Lack of impact by Rab4A on the expression of LAT1 in $CD4^+$ or $CD8^+$ T cells of age-matched female B6.TC, B6.TC/Rab4A^{Q72L}, and B6.TC/Rab4A^{Q72L} -KO mice or in Jurkat human $CD4^+$ T cells.** LAT1 is a transmembrane protein [2, 3], and thus its expression can be reliably detected by western blot analysis. Since mouse (45 kD) and human LAT1 (55 kD) migrate at different molecular weights, α-tubulin and β-actin were used as loading controls. **A**, Western blot analysis of LAT1 protein levels in $CD4^+$ and $CD8^+$ T cells of age-matched female B6.TC, B6.TC/Rab4A^{Q72L}, and B6.TC/Rab4A^{Q72L} -KO mice. Left panel, representative western blots; right panel, cumulative analysis. Overall two-way ANOVA p values are shown in the header of each figure panel, while Tukey's post-hoc test p values < 0.05 over brackets reflect comparison between experimental groups. **B**, Western blot analysis of LAT1 protein levels in Jurkat human $CD4^+$ T cell lines with altered expression of Rab4A. Left panel, representative western blots; right panel, cumulative analysis of four replicate experiments. Overall two-way ANOVA p values are shown in the header of each figure panel, while Tukey's post-hoc test p values < 0.05 over brackets reflect comparison between experimental groups.

Figure S16

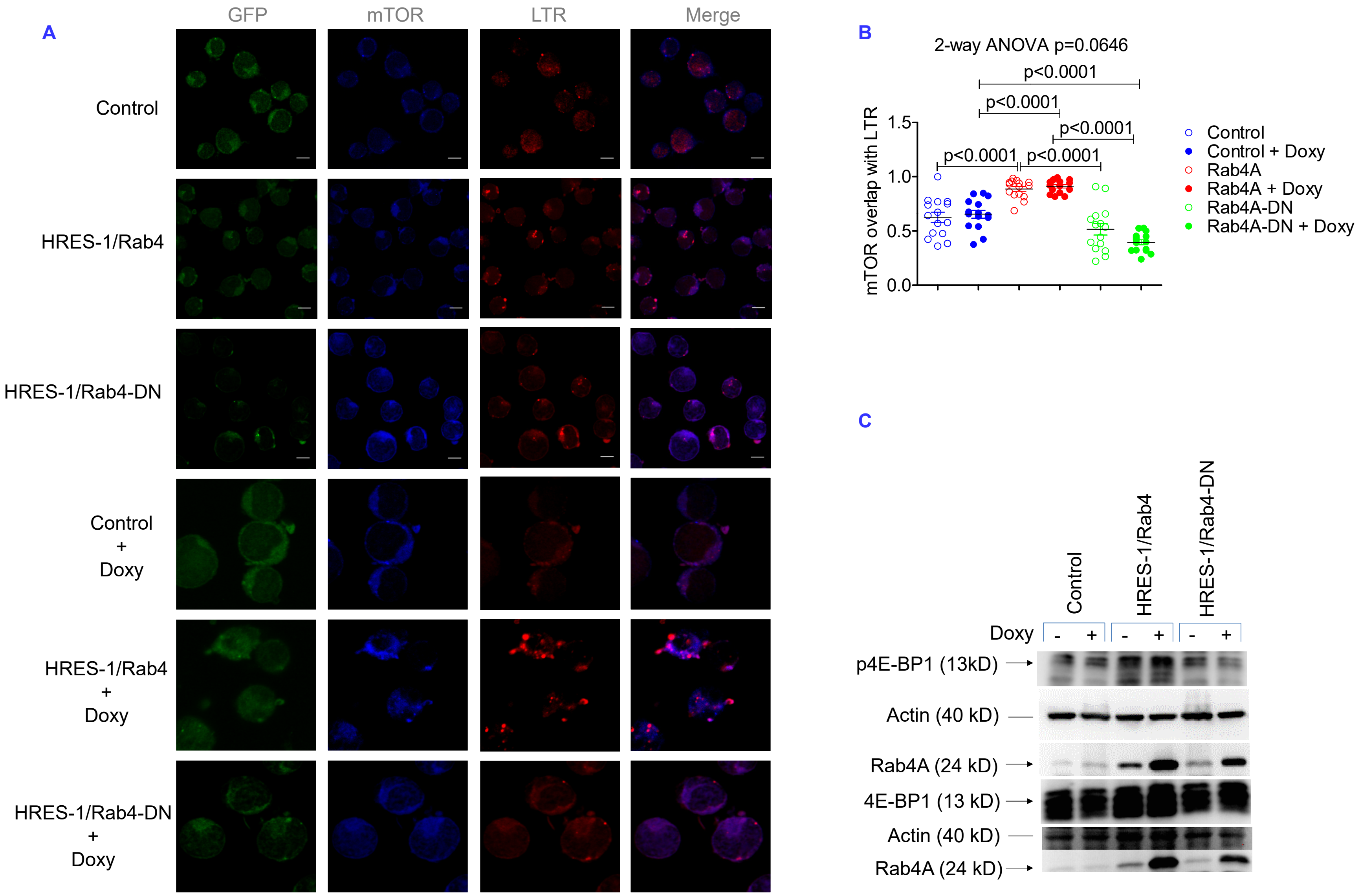
A
GFP
mTOR
LTR
Merge
Control
HRES-1/Rab4
HRES-1/Rab4-DN
Control + Doxy
HRES-1/Rab4 + Doxy
HRES-1/Rab4-DN + Doxy
B
2-way ANOVA p=0.0646
p<0.0001
p<0.0001
p<0.0001
p<0.0001
p<0.0001
mTOR overlap with LTR
1.5
1.0
0.5
0.0
Control
Control + Doxy
Rab4A
Rab4A + Doxy
Rab4A-DN
Rab4A-DN + Doxy
C
Control
HRES-1/Rab4
HRES-1/Rab4-DN
Doxy
- + - + - +
p4E-BP1 (13kD)
Actin (40 kD)
Rab4A (24 kD)
4E-BP1 (13 kD)
Actin (40 kD)
Rab4A (24 kD)

Figure S16. Effect of Rab4 on lysosomal traffic and activation of mTOR in Jurkat cells carrying AAV expression vector expressing green fluorescent protein (GFP) alone (Control) or wild-type or dominant-negative Rab4A^{S27N} (DN) Rab4A expressed from an open reading frame upstream of that of GFP. Cells were cultured with or without 1 ug/ml of doxycycline (Doxy). **A,** Representative confocal immunofluorescence microscopy images showing expression of GFP (green), mTOR (blue), and lysotracker red (LTR; red). **B,** Cumulative analysis of mTOR co-localization with LTR. Data points represent individuals cells analyzed in four independent experiments. Overall two-way ANOVA p values are shown in the header of each figure panel, while Tukey's post-hoc test p values < 0.05 over brackets reflect comparison between experimental groups. **C,** Western blot analysis of mTOR activity measured via phosphorylation of 4E-BP1. Wild-type Rab4A promoted mTOR activation, whereas dominant-negative Rab4A^{S27N} had the opposite effect. Expression of Rab4 and Rab4A^{S27N} by AAV vectors was moderately enhanced in the absence of doxycycline, which was attributed to leakage of the inducible promoter.

Figure S17

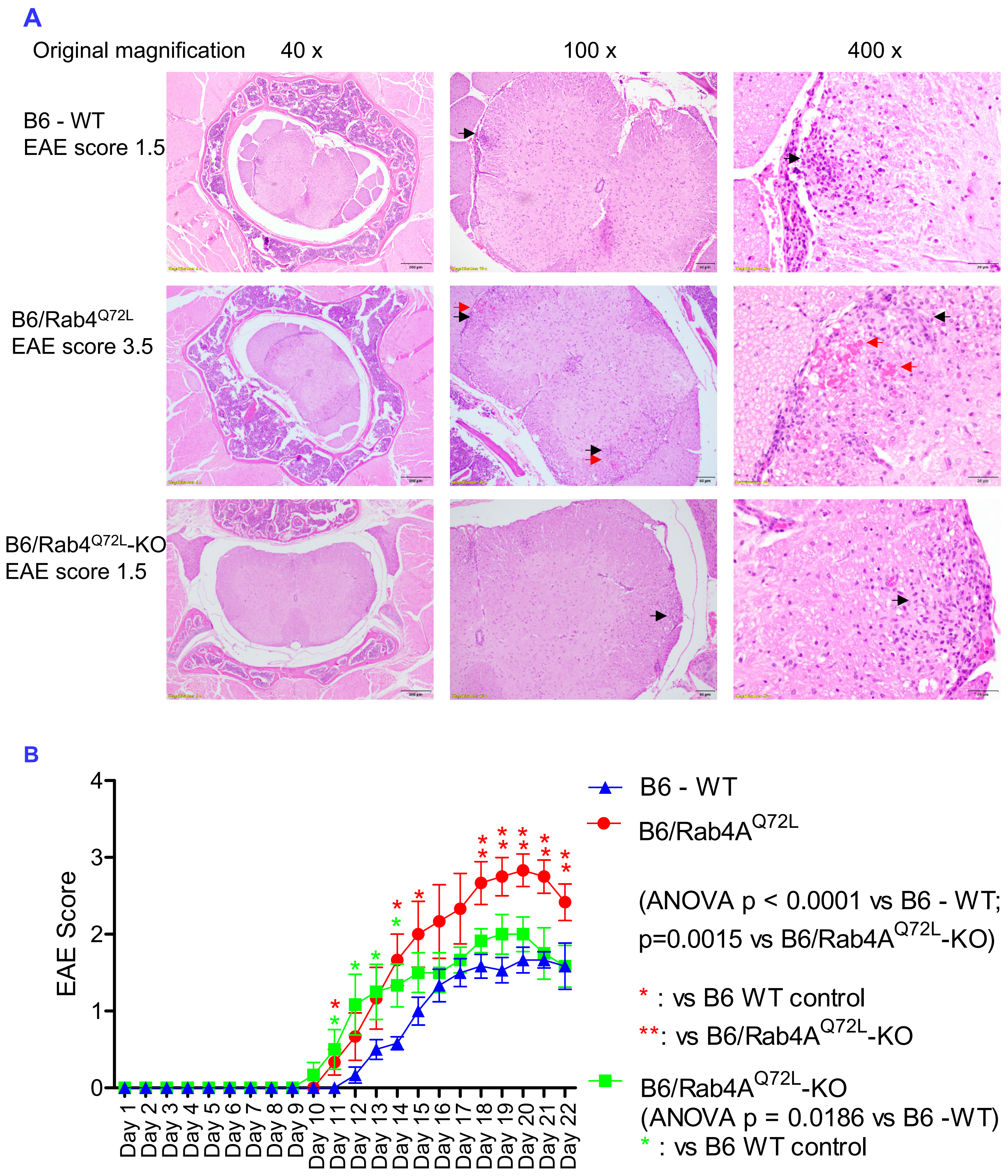

A
Original magnification 40 x
100 x
400 x
B6 - WT
EAE score 1.5
B6/Rab4Q72L
EAE score 3.5
B6/Rab4Q72L-KO
EAE score 1.5
B
EAE Score
B6 - WT
B6/Rab4AQ72L
(ANOVA p < 0.0001 vs B6 - WT;
p=0.0015 vs B6/Rab4AQ72L-KO)
* : vs B6 WT control
**: vs B6/Rab4AQ72L-KO
B6/Rab4AQ72L-KO
(ANOVA p = 0.0186 vs B6 -WT)
* : vs B6 WT control
Day 1
Day 2
Day 3
Day 4
Day 5
Day 6
Day 7
Day 8
Day 9
Day 10
Day 11
Day 12
Day 13
Day 14
Day 15
Day 16
Day 17
Day 18
Day 19
Day 20
Day 21
Day 22

Figure S17. Effect of Rab4A on MOG-induced EAE. For $MOG_{35–55}$-induced EAE, 10- to 12-wk-old female mice were immunized s.c. with 200 µl of an emulsion containing 800 µg of *Mycobacterium tuberculosis* H37Ra (Difco, Detroit, MI) and 200 µg $MOG_{35–55}$ distributed over three spots on the flank. Each mouse additionally received 200 ng pertussis toxin (PTx) (List Biological Laboratories, Campbell, CA) in 200 µl PBS i.p. on days 0 and 2 post immunization. **A**, Clinical scores of EAE. The data are plotted as the mean daily clinical score for all animals in a particular experimental group. The effect of genotypes were compared by repeated measures 2-way ANOVA: B6/Rab4A^{Q72L} vs WT, $p<0.0001$; B6/Rab4A^{Q72L}-KO vs WT, $p=0.0186$: B6/Rab4A^{Q72L} vs B6/Rab4A^{Q72L}-KO, $p=0.0015$. Comparisons were also made at each time points using two-t-test, asterisks reflect $p<0.05$. **B**, Spinal cord sections of mice immunized with MOG35-55 peptides. Spinal cords were stained with hematoxylin-eosin upon termination of experiment. Inflammatory infiltrates and intra-parenchymal hemorrhage are marked by black and red arrows, respectively. Magnification of original objectives and scale bars are embedded into each image using Olympus CellSense software.

Figure S18

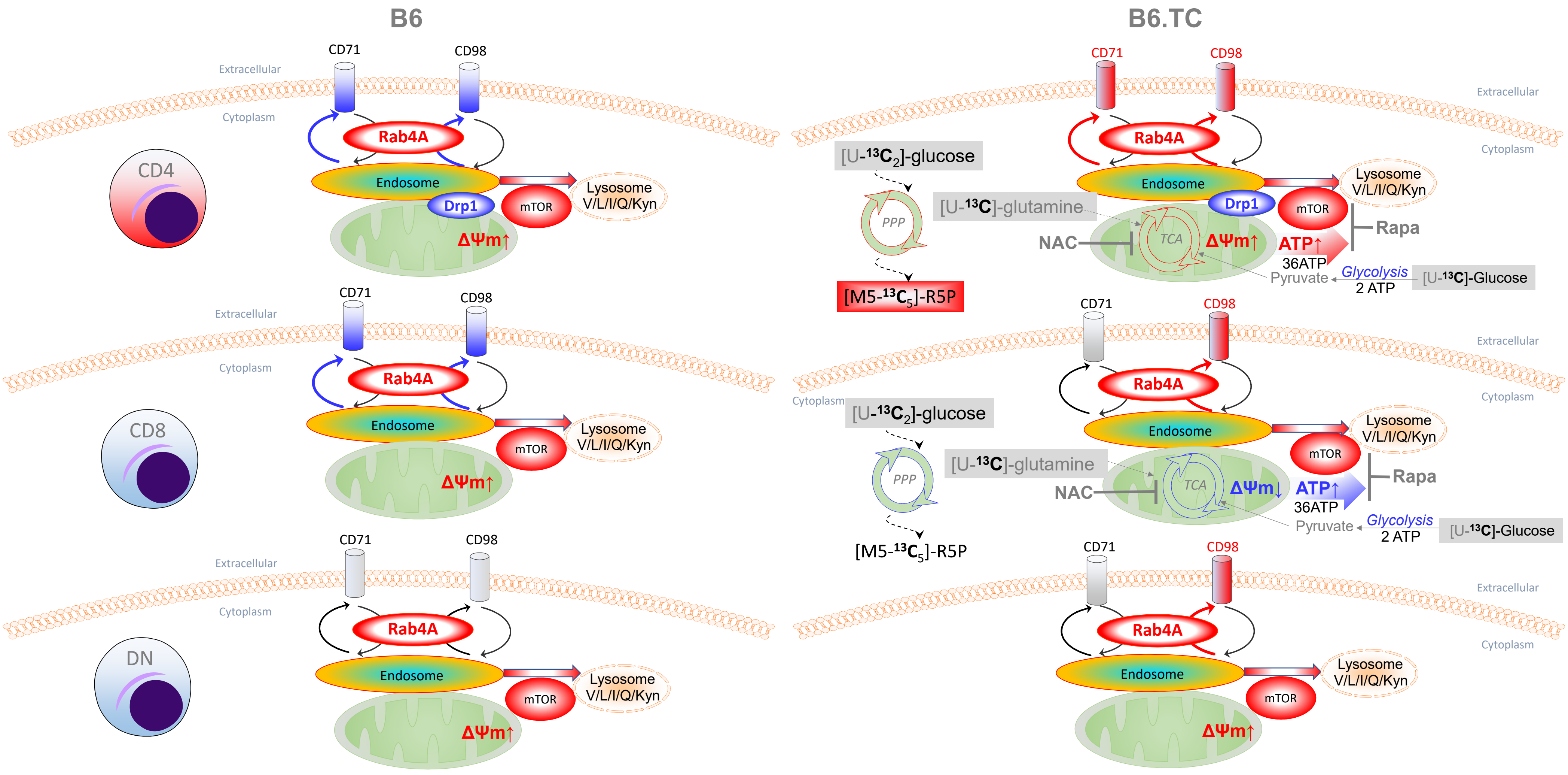
B6
B6.TC
CD71
CD98
Extracellular
Cytoplasm
CD4
CD8
DN
Rab4A
Endosome
Drp1
mTOR
Lysosome
V/L/I/Q/Kyn
ΔΨm↑
ΔΨm↓
[U-13C2]-glucose
PPP
[M5-13C5]-R5P
[U-13C]-glutamine
NAC
TCA
ATP↑
36ATP
Rapa
Glycolysis
2 ATP
Pyruvate
[U-13C]-Glucose

Figure S18. Rab4A-directed endosome traffic exerts cell-type specific control of mitochondrial metabolism via regulation of Drp1-mediated mitophagy, CD98 expression, and lysosomal localization of mTOR. 1) Rab4A elicits the depletion of mitophagy-initiator Drp1, and causes the accumulation of mitochondria in CD4 T cells of B6/Rab4A^{Q72L} mice and B6.TC/Rab4A^{Q72L} mice. Increased mitochondrial metabolism and metabolic flux through the TCA cycle is coupled with enhanced ATP production and increased metabolic flux through the PPP, which generates R5P, an essential precursor of *de novo* nucleotide biosynthesis. 2) Rab4A and SLE synergistically enhance the endocytic recycling of CD98, activate mTOR, and promote the expansion of CD4^{+} T cells in B6.TC/Rab4A^{Q72L} mice carrying constitutively active Rab4A alleles. CD98 mediates the uptake of KYN, which accumulates in CD8^{+} T cells and sera of B6.TC/Rab4A^{Q72L} mice and spreads mTOR activation to B cells and plasma cells. Inactivation of Rab4A in T cells blocks receptor recycling, mTOR activation, expansion of DN T cells, ABCs, and plasma cells, and GN in B6.TC/Rab4A^{Q72L}–KO mice. 3) Rab4A promotes the localization of mTOR to lysosomes. Pharmacological mTOR blockade with rapamycin and NAC abrogated CD98 expression, mTOR activation, and GN in B6.TC/Rab4A^{Q72L} mice.

Figure S19

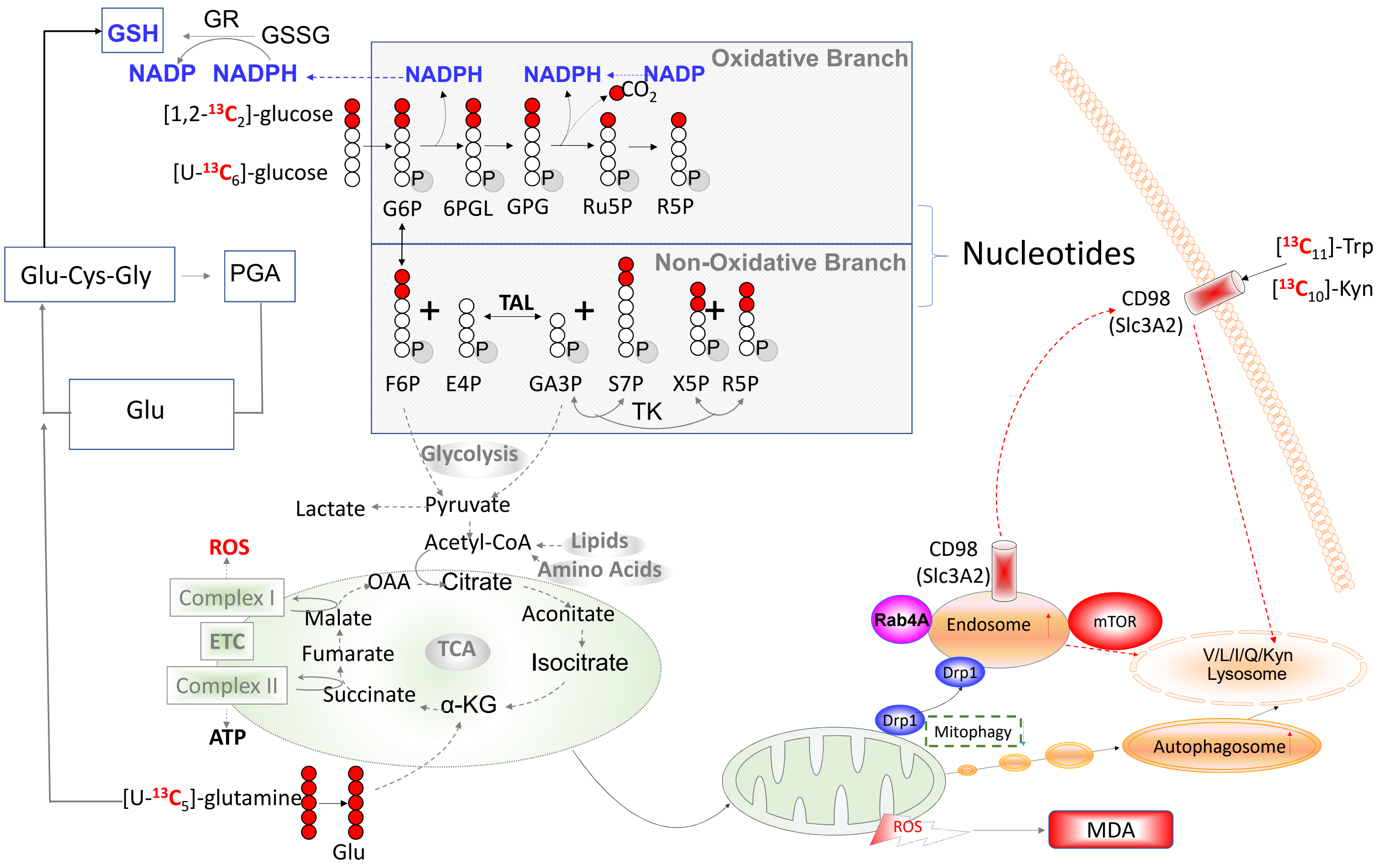

GSH
GR
GSSG
NADP
NADPH
Oxidative Branch
NADPH
NADPH
NADP
CO_2
[1,2-$^{13}C_2$]-glucose
[U-$^{13}C_6$]-glucose
P
G6P
6PGL
GPG
Ru5P
R5P
Glu-Cys-Gly
PGA
Non-Oxidative Branch
Nucleotides
TAL
F6P
E4P
GA3P
S7P
X5P
R5P
TK
Glu
[$^{13}C_{11}$]-Trp
[$^{13}C_{10}$]-Kyn
CD98
(Slc3A2)
Glycolysis
Lactate
Pyruvate
Acetyl-CoA
Lipids
Amino Acids
ROS
Complex I
ETC
Complex II
ATP
OAA
Citrate
Malate
Aconitate
Fumarate
TCA
Isocitrate
Succinate
α-KG
[U-$^{13}C_5$]-glutamine
Glu
CD98
(Slc3A2)
Rab4A
Endosome
mTOR
V/L/I/Q/Kyn
Lysosome
Drp1
Drp1
Mitophagy
Autophagosome
ROS
MDA

Figure S19. Schematic diagram of metabolic flux analyses through the pentose phosphate pathway (PPP), glycolysis, and the mitochondrial tricarboxylic acid (TCA) cycle.

Deuterated hydrogen (^{2}H) and carbon-13 ^{13}C atoms within the stable isotope-labelled compounds are marked in blue and red, respectively. We monitored the transfer of ^{2}H from [1-^{2}H]-glucose and [3-^{2}H]-glucose by PPP enzyme G6PD and 6PGD into NADPH, traced ^{13}C from [1,2-^{13}C]-glucose and [U-^{13}C]-glucose through the oxidative and non-oxidative branches of the PPP and glycolysis, and tracked ^{13}C from [U-^{13}C]-glutamine through the mitochondrial TCA cycle. Connections of metabolic flux are illustrated with antioxidant defenses such as regeneration of GSH form its oxidized form GSSG, neutralization of lipid peroxides, such as 4-hydroxynonenal (4-HNE), and the accumulation of oxidative stress-generating mitochondria and mTOR activation in $CD4^{+}$ T cells of lupus-prone B6.TC/$Rab4A^{Q72L}$ mice.

SUPPLEMENTARY REFERENCES

1. Lee,P.P. *et al.* A Critical Role for Dnmt1 and DNA Methylation in T Cell Development, Function, and Survival. *Immunity* **15**, 763-774 (2001).

2. Saito,Y. & Soga,T. Amino acid transporters as emerging therapeutic targets in cancer. *Cancer Sci.* **112**, 2958-2965 (2021).

3. Scalise,M., Galluccio,M., Console,L., Pochini,L., & Indiveri,C. The Human SLC7A5 (LAT1): The Intriguing Histidine/Large Neutral Amino Acid Transporter and Its Relevance to Human Health. *Front. Chem.* **6**, 243 (2018).
