# Supplementary material for: Rab4A-directed endosome traffic shapes pro-inflammatory mitochondrial metabolism in T cells via mitophagy, CD98 expression, and kynurenine-sensitive mTOR activation
Source: Nat Commun. 2024 Mar 22;15:2598. doi: 10.1038/s41467-024-46441-2 (PMC10960037; doi:10.1038/s41467-024-46441-2)
Supplement: Supplementary file 3 — Reporting Summary [file 41467_2024_46441_MOESM3_ESM.pdf]

nature portfolio

Corresponding author(s): . Perl, Andras
Last updated by author(s): 2024 2 3

Reporting Summary

Nature Portfolio wishes to improve the reproducibility of the work that we publish. This form provides structure for consistency and transparency in reporting. For further information on Nature Portfolio policies, see our Editorial Policies and the Editorial Policy Checklist.

Statistics

For all statistical analyses, confirm that the following items are present in the figure legend, table legend, main text, or Methods section.

n/a	Confirmed	
☐	☒	The exact sample size (*n*) for each experimental group/condition, given as a discrete number and unit of measurement
☐	☒	A statement on whether measurements were taken from distinct samples or whether the same sample was measured repeatedly
☐	☒	The statistical test(s) used AND whether they are one- or two-sided<br>*Only common tests should be described solely by name; describe more complex techniques in the Methods section.*
☐	☒	A description of all covariates tested
☐	☒	A description of any assumptions or corrections, such as tests of normality and adjustment for multiple comparisons
☐	☒	A full description of the statistical parameters including central tendency (e.g. means) or other basic estimates (e.g. regression coefficient) AND variation (e.g. standard deviation) or associated estimates of uncertainty (e.g. confidence intervals)
☐	☒	For null hypothesis testing, the test statistic (e.g. *F*, *t*, *r*) with confidence intervals, effect sizes, degrees of freedom and *P* value noted<br>*Give P values as exact values whenever suitable.*
☒	☐	For Bayesian analysis, information on the choice of priors and Markov chain Monte Carlo settings
☐	☒	For hierarchical and complex designs, identification of the appropriate level for tests and full reporting of outcomes
☒	☐	Estimates of effect sizes (e.g. Cohen's *d*, Pearson's *r*), indicating how they were calculated

Our web collection on statistics for biologists contains articles on many of the points above.

Software and code

Policy information about availability of computer code

Data collection	*Mass spectroscopy data were collected with the following softwares: Thermo Q Exactive Tune version2.9, Thermo TraceFinder version 4.1, and Thermo Scientific Xcalibur version 4.1; Flow Cytometry Data were acquired with the FACSDiva software version 6.0; Oxygraph Data were acquired with OxyTrace software; Seahorse Metabolic Analyzer data were acquired with Wave software version 2.6;*
Data analysis	*Metabolomic data were analyzed with Metaboanalyst version 5.0; Flow cytomtery data were analyzed with FlowJo software version 10.; RNAseq data were analyzed with RNA Express software; data analysis for statistical significance were analyzed with GraphPad Prism sofware version 5.0,*

For manuscripts utilizing custom algorithms or software that are central to the research but not yet described in published literature, software must be made available to editors and reviewers. We strongly encourage code deposition in a community repository (e.g. GitHub). See the Nature Portfolio guidelines for submitting code & software for further information.

Data

Policy information about availability of data

All manuscripts must include a data availability statement. This statement should provide the following information, where applicable:

- RNA-sequencing data will be deposited in the NCBI Gene Expression Omnibus database.

- For clinical datasets or third party data, please ensure that the statement adheres to our policy

All original data have been uploaded within Supplementary Materials.

Human research participants

Policy information about studies involving human research participants and Sex and Gender in Research.

Reporting on sex and gender	*Use the terms sex (biological attribute) and gender (shaped by social and cultural circumstances) carefully in order to avoid confusing both terms. Indicate if findings apply to only one sex or gender; describe whether sex and gender were considered in study design whether sex and/or gender was determined based on self-reporting or assigned and methods used. Provide in the source data disaggregated sex and gender data where this information has been collected, and consent has been obtained for sharing of individual-level data; provide overall numbers in this Reporting Summary. Please state if this information has not been collected. Report sex- and gender-based analyses where performed, justify reasons for lack of sex- and gender-based analysis.*
Population characteristics	*Describe the covariate-relevant population characteristics of the human research participants (e.g. age, genotypic information, past and current diagnosis and treatment categories). If you filled out the behavioural & social sciences study design questions and have nothing to add here, write "See above."*
Recruitment	*Describe how participants were recruited. Outline any potential self-selection bias or other biases that may be present and how these are likely to impact results.*
Ethics oversight	*Identify the organization(s) that approved the study protocol.*

Note that full information on the approval of the study protocol must also be provided in the manuscript.

Field-specific reporting

Please select the one below that is the best fit for your research. If you are not sure, read the appropriate sections before making your selection.

[X] Life sciences [ ] Behavioural & social sciences [ ] Ecological, evolutionary & environmental sciences

For a reference copy of the document with all sections, see nature.com/documents/nr-reporting-summary-flat.pdf

Life sciences study design

All studies must disclose on these points even when the disclosure is negative.

Sample size	Sample size was set at a minimum of 3 that has been found sufficient to detect accumulation of sedoheptulose 7-phosphate, a metabolic hallmark of transaldolase deficiency.
Data exclusions	Data from all technically well executed experiments were included.
Replication	Experiments were independently replicated to ensure reproducibility.
Randomization	Mice were allocated into groups according to genotype, age, and sex.
Blinding	Mice were serially numbered and experiments were carried out without awareness of genotypes. Pathologists were reviewing histology were blinded to mouse genotype or treatment groups.

Behavioural & social sciences study design

All studies must disclose on these points even when the disclosure is negative.

Study description	*Briefly describe the study type including whether data are quantitative, qualitative, or mixed-methods (e.g. qualitative cross-sectional, quantitative experimental, mixed-methods case study).*
Research sample	*State the research sample (e.g. Harvard university undergraduates, villagers in rural India) and provide relevant demographic information (e.g. age, sex) and indicate whether the sample is representative. Provide a rationale for the study sample chosen. For studies involving existing datasets, please describe the dataset and source.*
Sampling strategy	*Describe the sampling procedure (e.g. random, snowball, stratified, convenience). Describe the statistical methods that were used to predetermine sample size OR if no sample-size calculation was performed, describe how sample sizes were chosen and provide a rationale for why these sample sizes are sufficient. For qualitative data, please indicate whether data saturation was considered, and what criteria were used to decide that no further sampling was needed.*

Data collection	*Provide details about the data collection procedure, including the instruments or devices used to record the data (e.g. pen and paper, computer, eye tracker, video or audio equipment) whether anyone was present besides the participant(s) and the researcher, and whether the researcher was blind to experimental condition and/or the study hypothesis during data collection.*
Timing	*Indicate the start and stop dates of data collection. If there is a gap between collection periods, state the dates for each sample cohort.*
Data exclusions	*If no data were excluded from the analyses, state so OR if data were excluded, provide the exact number of exclusions and the rationale behind them, indicating whether exclusion criteria were pre-established.*
Non-participation	*State how many participants dropped out/declined participation and the reason(s) given OR provide response rate OR state that no participants dropped out/declined participation.*
Randomization	*If participants were not allocated into experimental groups, state so OR describe how participants were allocated to groups, and if allocation was not random, describe how covariates were controlled.*

Ecological, evolutionary & environmental sciences study design

All studies must disclose on these points even when the disclosure is negative.

Study description	*Briefly describe the study. For quantitative data include treatment factors and interactions, design structure (e.g. factorial, nested, hierarchical), nature and number of experimental units and replicates.*
Research sample	*Describe the research sample (e.g. a group of tagged Passer domesticus, all Stenocereus thurberi within Organ Pipe Cactus National Monument), and provide a rationale for the sample choice. When relevant, describe the organism taxa, source, sex, age range and any manipulations. State what population the sample is meant to represent when applicable. For studies involving existing datasets, describe the data and its source.*
Sampling strategy	*Note the sampling procedure. Describe the statistical methods that were used to predetermine sample size OR if no sample-size calculation was performed, describe how sample sizes were chosen and provide a rationale for why these sample sizes are sufficient.*
Data collection	*Describe the data collection procedure, including who recorded the data and how.*
Timing and spatial scale	*Indicate the start and stop dates of data collection, noting the frequency and periodicity of sampling and providing a rationale for these choices. If there is a gap between collection periods, state the dates for each sample cohort. Specify the spatial scale from which the data are taken*
Data exclusions	*If no data were excluded from the analyses, state so OR if data were excluded, describe the exclusions and the rationale behind them, indicating whether exclusion criteria were pre-established.*
Reproducibility	*Describe the measures taken to verify the reproducibility of experimental findings. For each experiment, note whether any attempts to repeat the experiment failed OR state that all attempts to repeat the experiment were successful.*
Randomization	*Describe how samples/organisms/participants were allocated into groups. If allocation was not random, describe how covariates were controlled. If this is not relevant to your study, explain why.*
Blinding	*Describe the extent of blinding used during data acquisition and analysis. If blinding was not possible, describe why OR explain why blinding was not relevant to your study.*

Did the study involve field work? ☐ Yes ☒ No

Field work, collection and transport

Field conditions	*Describe the study conditions for field work, providing relevant parameters (e.g. temperature, rainfall).*
Location	*State the location of the sampling or experiment, providing relevant parameters (e.g. latitude and longitude, elevation, water depth).*
Access & import/export	*Describe the efforts you have made to access habitats and to collect and import/export your samples in a responsible manner and in compliance with local, national and international laws, noting any permits that were obtained (give the name of the issuing authority, the date of issue, and any identifying information).*
Disturbance	*Describe any disturbance caused by the study and how it was minimized.*

Reporting for specific materials, systems and methods

We require information from authors about some types of materials, experimental systems and methods used in many studies. Here, indicate whether each material, system or method listed is relevant to your study. If you are not sure if a list item applies to your research, read the appropriate section before selecting a response.

Materials & experimental systems

n/a	Involved in the study	
☐	☒	Antibodies
☐	☒	Eukaryotic cell lines
☒	☐	Palaeontology and archaeology
☐	☒	Animals and other organisms
☒	☐	Clinical data
☒	☐	Dual use research of concern

Methods

n/a	Involved in the study	
☒	☐	ChIP-seq
☐	☒	Flow cytometry
☒	☐	MRI-based neuroimaging

Antibodies

Antibodies used

All antibodies used in the study are referenced by supplier name and catalog number and dilution on page 5.

Validation

All unique antibodies are validated within the manuscript by assessment of molecular weight.

Eukaryotic cell lines

Policy information about cell lines and Sex and Gender in Research

Cell line source(s)

Cell lines expressing genetically altered alleles of Rab4A were validated by DNA sequencing and western blot analyses. All other cell lines are referenced in the manuscript.

Authentication

All cell lines were validated for expression of Rab4A by western blot using actin loading control for each membrane.

Mycoplasma contamination

All cell lines tested negative for mycoplasma contamination.

Commonly misidentified lines
(See ICLAC register)

None.

Palaeontology and Archaeology

Specimen provenance

Provide provenance information for specimens and describe permits that were obtained for the work (including the name of the issuing authority, the date of issue, and any identifying information). Permits should encompass collection and, where applicable, export.

Specimen deposition

Indicate where the specimens have been deposited to permit free access by other researchers.

Dating methods

If new dates are provided, describe how they were obtained (e.g. collection, storage, sample pretreatment and measurement), where they were obtained (i.e. lab name), the calibration program and the protocol for quality assurance OR state that no new dates are provided.

☐ Tick this box to confirm that the raw and calibrated dates are available in the paper or in Supplementary Information.

Ethics oversight

Identify the organization(s) that approved or provided guidance on the study protocol, OR state that no ethical approval or guidance was required and explain why not.

Note that full information on the approval of the study protocol must also be provided in the manuscript.

Animals and other research organisms

Policy information about studies involving animals; ARRIVE guidelines recommended for reporting animal research, and Sex and Gender in Research

Laboratory animals

C57BL/6 mice with wild-type (WT), transaldolase-deleted (TALKO), aldose reductase-deleted (ARKO), or dual transaldolase and aldose reductase-deleted (DKO) alleles were used. Mice were matched for age and gender in each experiment, as reported in figure legends. The C57BL/6 background strain originated from Jackson Laboratories in ~2000.

Antibody reagents used for western blot analyses. Rabbit monoclonal Rab4A antibody was obtained from Abcam (Cat No. ab108974; Cambridge, MA). Mouse monoclonal p70S6 kinase (p70S6K) (Catalog No. sc-8418), and mouse monoclonal phospho-p70S6K antibodies (Catalog No. sc-8416) were obtained from Santa Cruz Biotechnology (Santa Cruz, CA). Rabbit monoclonal p70S6K (Catalog No. 2708), AKT (Catalog No. 4685), rabbit polyclonal phospho-p70S6K (Catalog No. 9205), and phospho-AKT (Catalog No. 4058) were purchased from Cell Signaling Technology. β-actin mouse monoclonal antibody (Catalog No. Mab1501R) and anti-Cre recombinase rabbit polyclonal antibody were obtained from Sigma Millipore (Burlington, MA, Cat No 69050-3). Primary antibodies were employed at 1000-fold dilutions with the exception of β-actin antibody which was employed at 5000-fold dilution. Secondary antibodies conjugated to horse radish peroxidase (HRP) were employed at 1:20000 dilution.

Antibody reagents used in flow cytometry studies.

Antibody target	Fluorochrome	Company	Cat. No.
CD3	BUV737	BD	564618
CD3	BV711	BD	563123
CD4	BUV496	BD	564667
CD4	PerCP-Cy5.5	BD	550954
CD8	APC-Cy7	Biolegend	100714
CD11b	PerCP-Cy5.5	BioLegend	101228
CD11b	BUV395	BD	563553
CD11c	BUV395	BD	744180
CD11c	AF488	Biolegend	117311
CD11c	PE-CF594	BD	562464
CD11c	BV605	BD	563057
CD19	APC-Cy7	BD	557655
CD19	AF700	Biolegend	115528
CD19	APC-R700	BD	565473
CD19	PerCP-Cy5.5	BD	551001
CD25	BUV395	BD	564022
CD38	PE-Cy7	Biolegend	102718
CD38	BV786	BD	740887
CD47	BV421	Biolegend	127527
CD51	BV650	BD	740546
CD61	BV510	BD	740117
CD68	PE-Cy7	Biolegend	137016
CD71	PE-Cy7	Biolegend	113812
CD98	PE	Invitrogen	12-0981-83
CD152	PE-Dazzle594	Biolegend	106318
D2DR	FITC	Alomone	ADR-002-F
DRD2	Alexa647	Santa Cruz	SC-5303
GLUT1	AF700	R&D	FAB1418N
GLUT4	AF647	R&D	FAB86541R
SERT	FITC	Alomone	AMT-004-F
CCR4	PE-Cy7	Biolegend	131214
CCR6	AF647	BD	557976
Gr1	PE-CF594	BD	562700
FoxP3	PerCP-Cy5.5	eBioscience	45-5773-82
Helios	AF647	Biolegend	137218
IFNy	AF647	Biolegend	505814
IL4	PE-Cy7	Biolegend	504118
IL9	PerCP-Cy5.5	Biolegend	514112
IL17a	PE/Dazzle594	Biolegend	506938
IL17a	AF488	Biolegend	506910
IL21	PE	eBioscience	12-7213-82
MTG	516nm	Invitrogen	M7514
pAKT	PE	BD	560378
pS6RP	AF488	Cell Signaling	4803S
TMRM	585nm	Invitrogen	M20036
FC block		BD	553142
FixPerm concentrate (10x)		eBioscience	00-5123-43
Fix Perm diluent		eBioscience	00-5223-56
Permeabilization buffer (10x)		eBioscience	00-8333-56

Isotype controls	Fluorochrome	Company	Catalogue No
Hamster IgG1	BV605	BD	563054
Hamster IgG1	BV510	BD	563197
Rat IgG1	BV650 º	BD	563848
Hamster IgG1	BV711	BD	563128
Rat IgG2a	BV786	BD	563335
Mouse IgG1	PE	BD	556650
Rat IgG2a	Brilliant Violet 421	Biolegend	400535
Mouse IgG2	Alexa Fluor 647	Biolegend	400234

Wild animals	*Provide details on animals observed in or captured in the field; report species and age where possible. Describe how animals were caught and transported and what happened to captive animals after the study (if killed, explain why and describe method; if released, say where and when) OR state that the study did not involve wild animals.*
Reporting on sex	*Sex was considered in study design. Mice of different genotypes were matched for age, and gender, typically 20-week-old females.*
Field-collected samples	*NIA*
Ethics oversight	*This research has been reviewed, approved, and carried out in compliance with the policies of the Institutional Animal Care and Use Committee of the State University of New York, Upstate Medical University, Syracuse, NY USA*

Note that full information on the approval of the study protocol must also be provided in the manuscript.

Clinical data

Policy information about clinical studies
All manuscripts should comply with the ICMJE guidelines for publication of clinical research and a completed CONSORT checklist must be included with all submissions.

Clinical trial registration	*ClinicalTrials.gov Identifier: NCT00779194*
Study protocol	*https:llpubmed.ncbi.nlm.nih.govl29551338l*
Data collection	*Describe the settings and locales of data collection, noting the time periods of recruitment and data collection.*
Outcomes	*Describe how you pre-defined primary and secondary outcome measures and how you assessed these measures.*

Dual use research of concern

Policy information about dual use research of concern

Hazards

Could the accidental, deliberate or reckless misuse of agents or technologies generated in the work, or the application of information presented in the manuscript, pose a threat to:

No	Yes	
X	☐	Public health
X	☐	National security
X	☐	Crops and/or livestock
X	☐	Ecosystems
X	☐	Any other significant area

Experiments of concern

Does the work involve any of these experiments of concern:

No	Yes	
X	☐	Demonstrate how to render a vaccine ineffective
X	☐	Confer resistance to therapeutically useful antibiotics or antiviral agents
X	☐	Enhance the virulence of a pathogen or render a nonpathogen virulent
X	☐	Increase transmissibility of a pathogen
X	☐	Alter the host range of a pathogen
X	☐	Enable evasion of diagnostic/detection modalities
X	☐	Enable the weaponization of a biological agent or toxin
X	☐	Any other potentially harmful combination of experiments and agents

ChIP-seq

Data deposition

☐ Confirm that both raw and final processed data have been deposited in a public database such as GEO.

☐ Confirm that you have deposited or provided access to graph files (e.g. BED files) for the called peaks.

Data access links<br>*May remain private before publication.*	*For "Initial submission" or "Revised version" documents, provide reviewer access links. For your "Final submission" document, provide a link to the deposited data.*
Files in database submission	*Provide a list of all files available in the database submission.*
Genome browser session<br>(e.g. UCSC)	*Provide a link to an anonymized genome browser session for "Initial submission" and "Revised version" documents only, to enable peer review. Write "no longer applicable" for "Final submission" documents.*

Methodology

Replicates	*Describe the experimental replicates, specifying number, type and replicate agreement.*
Sequencing depth	*Describe the sequencing depth for each experiment, providing the total number of reads, uniquely mapped reads, length of reads and whether they were paired- or single-end.*
Antibodies	*Describe the antibodies used for the ChIP-seq experiments; as applicable, provide supplier name, catalog number, clone name, and lot number.*
Peak calling parameters	*Specify the command line program and parameters used for read mapping and peak calling, including the ChIP, control and index files used.*
Data quality	*Describe the methods used to ensure data quality in full detail, including how many peaks are at FDR 5% and above 5-fold enrichment.*
Software	*Describe the software used to collect and analyze the ChIP-seq data. For custom code that has been deposited into a community repository, provide accession details.*

Flow Cytometry

Plots

Confirm that:

☒ The axis labels state the marker and fluorochrome used (e.g. CD4-FITC).

☒ The axis scales are clearly visible. Include numbers along axes only for bottom left plot of group (a 'group' is an analysis of identical markers).

☒ All plots are contour plots with outliers or pseudocolor plots.

☒ A numerical value for number of cells or percentage (with statistics) is provided.

Methodology

Sample preparation	*Samples were resuspended in phosphate buffered saline, tissue culture medium, or specified in Supplemental aMedthods and figure legends for each experiment.*
Instrument	*Data were acquired on a Becton-Dickinson LSR II flow cytometer.*
Software	*FASCDIva Software was used for data collection.*
Cell population abundance	*Typically 10,000 live cells or viable organelles, such as mitochondria, were analyzed in each sample.*
Gating strategy	*Viable cells and organelles were initially gated by FSC/SSC analysis.*

☒ Tick this box to confirm that a figure exemplifying the gating strategy is provided in the Supplementary Information.

Magnetic resonance imaging

Experimental design

Design type	*Indicate task or resting state; event-related or block design.*

Design specifications	*Specify the number of blocks, trials or experimental units per session and/or subject, and specify the length of each trial or block (if trials are blocked) and interval between trials.*
Behavioral performance measures	*State number and/or type of variables recorded (e.g. correct button press, response time) and what statistics were used to establish that the subjects were performing the task as expected (e.g. mean, range, and/or standard deviation across subjects).*

Acquisition

Imaging type(s)	*Specify: functional, structural, diffusion, perfusion.*
Field strength	*Specify in Tesla*
Sequence & imaging parameters	*Specify the pulse sequence type (gradient echo, spin echo, etc.), imaging type (EPI, spiral, etc.), field of view, matrix size, slice thickness, orientation and TE/TR/flip angle.*
Area of acquisition	*State whether a whole brain scan was used OR define the area of acquisition, describing how the region was determined.*

Diffusion MRI ☐ Used ☐ Not used

Preprocessing

Preprocessing software	*Provide detail on software version and revision number and on specific parameters (model/functions, brain extraction, segmentation, smoothing kernel size, etc.).*
Normalization	*If data were normalized/standardized, describe the approach(es): specify linear or non-linear and define image types used for transformation OR indicate that data were not normalized and explain rationale for lack of normalization.*
Normalization template	*Describe the template used for normalization/transformation, specifying subject space or group standardized space (e.g. original Talairach, MNI305, ICBM152) OR indicate that the data were not normalized.*
Noise and artifact removal	*Describe your procedure(s) for artifact and structured noise removal, specifying motion parameters, tissue signals and physiological signals (heart rate, respiration).*
Volume censoring	*Define your software and/or method and criteria for volume censoring, and state the extent of such censoring.*

Statistical modeling & inference

Model type and settings	*Specify type (mass univariate, multivariate, RSA, predictive, etc.) and describe essential details of the model at the first and second levels (e.g. fixed, random or mixed effects; drift or auto-correlation).*
Effect(s) tested	*Define precise effect in terms of the task or stimulus conditions instead of psychological concepts and indicate whether ANOVA or factorial designs were used.*

Specify type of analysis: ☐ Whole brain ☐ ROI-based ☐ Both

Statistic type for inference (See Eklund et al. 2016)	*Specify voxel-wise or cluster-wise and report all relevant parameters for cluster-wise methods.*
Correction	*Describe the type of correction and how it is obtained for multiple comparisons (e.g. FWE, FDR, permutation or Monte Carlo).*

Models & analysis

n/a	Involved in the study
☐	☐ Functional and/or effective connectivity
☐	☐ Graph analysis
☐	☐ Multivariate modeling or predictive analysis

Functional and/or effective connectivity	*Report the measures of dependence used and the model details (e.g. Pearson correlation, partial correlation, mutual information).*
Graph analysis	*Report the dependent variable and connectivity measure, specifying weighted graph or binarized graph, subject- or group-level, and the global and/or node summaries used (e.g. clustering coefficient, efficiency, etc.).*
Multivariate modeling and predictive analysis	*Specify independent variables, features extraction and dimension reduction, model, training and evaluation metrics.*
